# A comprehensive analysis of perturbation methods in explainable AI feature attribution validation for neural time series classifiers

**DOI:** 10.1038/s41598-025-09538-2

**Published:** 2025-07-22

**Authors:** Ilija Šimić, Eduardo Veas, Vedran Sabol

**Affiliations:** 1 Graz University of Technology, Graz, Austria; 2 Know Center Research GmbH, Graz, Austria

**Keywords:** Explainable AI, Deep learning, Attribution methods, Evaluation, Time series, DDS, PES, CMI, Computer science, Statistics

## Abstract

In domains where AI model predictions have significant consequences, such as industry, medicine, and finance, the need for explainable AI (XAI) is of utmost importance. However, ensuring that explanation methods provide faithful and trustworthy explanations requires rigorous validation. Feature attribution methods (AMs) are among the most prevalent explanation methods, as they identify decisive aspects that influence model predictions through feature importance estimates. Evaluating the correctness of AMs is typically done by systematically perturbing features according to their estimated importance and measuring the impact on the classifier’s performance. This paper extends our previous work which revealed flaws in the most commonly used metric for validating AMs when applied to time series data. In this work we introduce a novel metric, the Consistency-Magnitude-Index, which facilitates a faithful assessment of feature importance attribution. Additionally, we introduce an adapted methodology for robust faithfulness evaluation, leveraging a set of diverse perturbation methods. Our work includes an extended evaluation of AMs on time series data, that presents the influence and importance of perturbation methods and region size selection in relation to dataset and model characteristics. Based on the results of our extensive evaluation, we provide guidelines for future AM faithfulness assessments. Finally, we demonstrate our methodology through a concrete multivariate time series example.

## Introduction

The black-box nature of deep learning (DL) models is a major obstacle that hinders their wide-spread application in high-stakes domains, such as industry, medicine or finance. Given that in these domains mispredictions can lead to severe outcomes, it is necessary that each model prediction can be explained. This in turn increased the attention towards the field of eXplainable AI (XAI), which revolves around methods that try to explain model predictions.

In this paper we focus on *XAI methods that explain model predictions by identifying the most influential input features*, also referred to as *feature Attribution Methods (AM)*. AMs have gained popularity due to their capability to provide understandable explanations for individual model predictions. For instance, these methods can be used to verify whether a model’s decisions align with human intuition or rely on spurious correlations^1^.

**Fig. 1:**
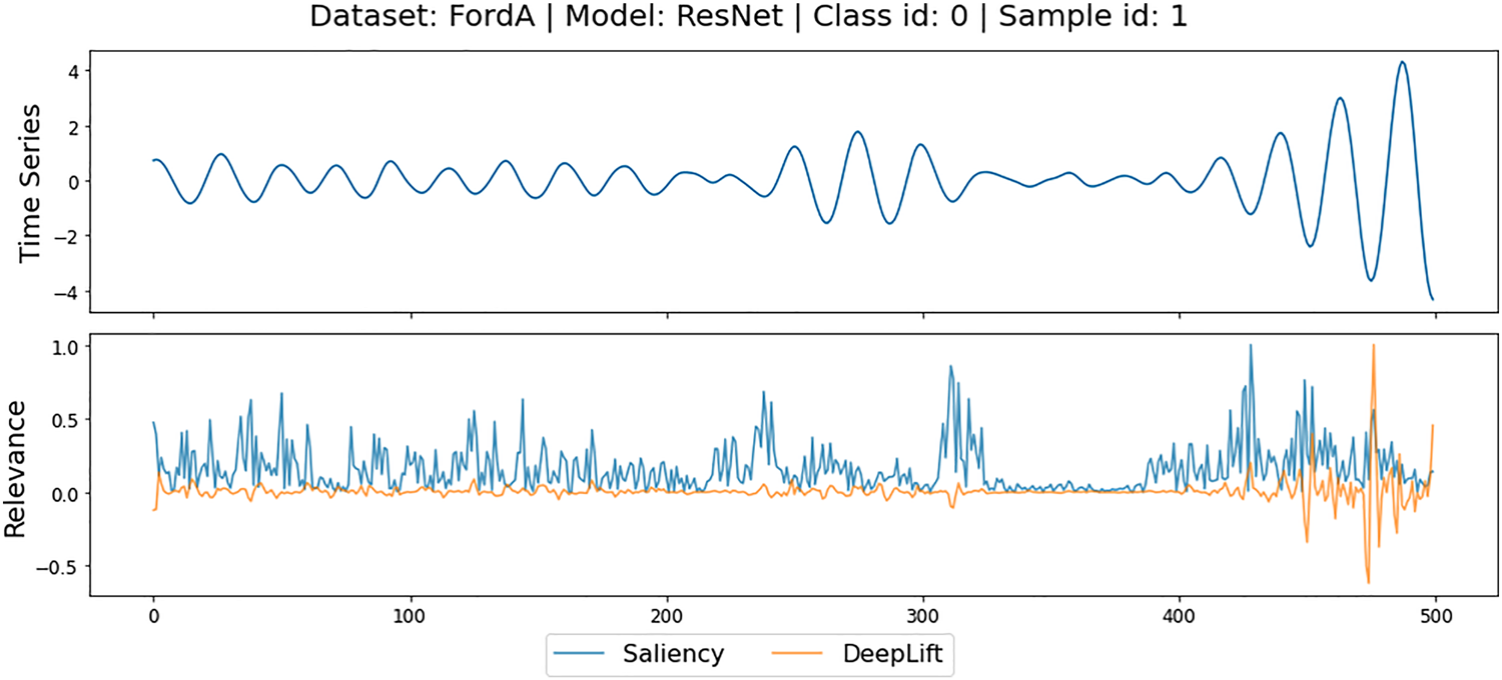
Different attribution methods (AMs) identifying different time steps of the same time series (upper curve) as important, given the same model prediction.

While numerous AMs have been introduced, selecting an AM which faithfully explains a model is nontrivial due to frequently disagreeing explanations produced by different methods for the same prediction (Fig. 1). In this context, *faithfulness*^2^, also known as *fidelity*, refers to the ability of an AM to identify input features which were *truly* crucial for the model prediction. The most popular approach for estimating an AMs faithfulness is *region perturbation*^3^, which assesses the quality of an explanation by measuring the impact of perturbing features based on their relevance.

Most evaluations of AMs using perturbation-based approaches have been conducted with models trained on image^3,4^ and text data^5,6^, whereas time series data has been comparatively neglected. In this paper, we investigate the faithfulness of AMs for neural time series classification models, given the importance of this data type in high-stakes domain, as well as the fact that deep learning time series classification models have become comparable to the state of the art^7,8^.

Perturbation Methods (PMs) in perturbation-based AM faithfulness evaluations are in previous literature often chosen arbitrarily^3,4,9^. This observation has prompted us to investigate the effects of PM selection on the outcomes of AM faithfulness evaluation, as well as the influence of the size of the perturbed regions. Moreover, our motivation stems from initial experiments where we observed the potentially devastating consequences of selecting an unsuitable PM. This resulted in abrupt changes in a classifiers predicted class probability from 1 to 0 after minimal perturbations, regardless of the features’ actual relevance, due to shifts in the data distribution^10^. Similar effects have been noted in previous work involving images^11^ and text^12^.

In this paper, we present the substantial impact of PM selection on the faithfulness evaluation of AMs, *emphasizing the importance of considering multiple PMs rather than relying solely on one*. Furthermore, we demonstrate that region size selection has a comparatively lesser impact on AM faithfulness evaluation, although with differences in PM suitability. We also show that the metric which is commonly used in region perturbation, the *Area Under Perturbation Curve (AUPC)* in the *most relevant first (MoRF)* order, should not be used by itself to estimate the faithfulness of AMs, and may actually lead to wrong conclusions. Moreover, this metric fails to provide insight into the *extent* to which relevant and irrelevant features can be separated, and how *consistently*. Therefore, we re-introduce two metrics which improve the deficiencies of the $AUPC_{MoRF}$ metric: (i) the *Decaying Degradation Score (DDS)*—quantifies the degree of separation between relevant and irrelevant features, and (ii) the *Perturbation Effect Size (PES)*—measures how *consistently* an AM can distinguish important from unimportant features. Furthermore, we combine these two metrics into a new metric, the *Consistency-Magnitude-Index (CMI)*, to streamline the identification of AMs which most consistently separate important from unimportant features to the greatest extent.

Based on our extensive AM faithfulness evaluation, we provide guidelines on how to evaluate the faithfulness of AMs with neural time series classification models, as well as how to select the most faithful AM for a specific dataset and model.

This paper is an extension of our previous work, which was published at the *International Conference on Information & Knowledge Management*^13^. In summary, we extend the conference paper by:

1. Combining the previously introduced *PES* and *DDS* into a *novel metric*—the *Consistency-Magnitude-Index*. (Section “Consistency-Magnitude-Index (*CMI*)”)
2. Introducing a *robust AM faithfulness* evaluation methodology—that employs a set of PMs to identify the most faithful AMs for neural time series classification models. (Section “Methodology for AM faithfulness estimation”)
3. Extending our AM faithfulness experiments for neural time series classification models (Section “Experimental setup”), by:
  Investigating a broader spectrum of DL model architectures (from 2 to 5)
  Investigating a wider range of AMs (from 9 to 12),
  Including additional PMs, many of which are time series specific (from 8 to 23),
4. Providing a comprehensive examination of experimental results across different dataset types with distinct characteristics (Section “Results”):
  1 Binary imbalanced dataset (anomaly detection),
  2 Binary balanced datasets,
  2 Multiclass datasets
  in combination with the investigated model architectures.
5. Providing guidelines for future AM faithfulness evaluations. (Section “Summary of findings & guidelines for AM faithfulness evaluations”)
6. Demonstrating the applicability of our methodology on multivariate time series data. (Section “Practical example: AM faithfulness evaluation for a multivariate time series classifier”)

Throughout this paper, references to *“our previous paper”* or *“our previous results”* denote findings from the conference paper that we extend.

While the choice of PM might be more straightforward or less impactful in other domains (e.g., image classification), our results highlight the sensitivity of time series classifiers to PM selection. Also, our findings expose potential flaws in previous evaluations of AMs on time series data. These flaws stem from the arbitrary choice and dependence on a single PM and the use of $AUPC_{MoRF}$ as the primary evaluation metric. It is likely that similar issues affect AM evaluations in other data types as well.

We also reveal that there is no universally optimal PM nor AM for all investigated model architectures and datasets. Most importantly, we show that data properties are not the sole factor influencing the optimal PM selection; what the model has learned to rely on must also be considered. Similarly, the model architecture is not the sole determining factor for AM faithfulness, but the data characteristics as well. However, we offer a guided blueprint for selecting a set of PMs that can ensure a robust AM evaluation. We believe this approach will enable researchers and practitioners to compare, validate and select the most reliable AMs for their specific requirements.

The remainder of this paper is structured as follows. Section “Background” introduces relevant concepts and discusses related work. Section “Problem statement” formally defines the problem statement of this paper. In Section “Methods for faithfulness estimation” we re-introduce the Perturbation Effect Size (*PES*) and the Decaying Degradation Score (*DDS*) metrics, and introduce the novel Consistency-Magnitude Index (*CMI*). This section also depicts the adapted faithfulness evaluation methodology. Section “Experiments” describes the experimental setup, discusses the results, and provides guidelines for future AM evaluations based on the results. The “Practical example: AM faithfulness evaluation for a multivariate time series classifier” section demonstrates how our methodology can be transferred to a multivariate time series dataset. The “Limitations and future work” section discusses the limitations of the proposed approach and provides an outlook on future work. Finally, Section “Conclusion” summarizes the main findings of the paper.

## Background

Within machine learning, deep learning (DL) refers to a set of algorithms that use layers of subsequent non-linear computational units (neurons) enabling them to learn hierarchical representations of the data. However, due to their complexity, DL models are often referred to as black boxes, or black-box models. This implies that the relationship between an input, and a model’s prediction cannot be easily understood by simply observing the model’s internals. The desire to understand these complex models led to the development of many approaches for explaining what the model has learned. Depending on the scope, explanations can be either global or local^14^. While global explanations try to identify the general decision-making strategies of the model, local explanations explain why a model made a specific prediction for a single input. Further, they are different types of local explanations, such as: feature attribution methods^15,16^, counterfactual explanations^17^, surrogate models^18^, concept-based approaches^19^.

In this work we focus on attribution methods (AMs), which are local explanation methods that explain a model’s prediction by estimating the relevance of each input feature for a single model prediction. Many AMs have been introduced in recent years, which utilize different approaches to compute feature relevance, e.g., AMs relying on gradients^20–23^, information backpropagation^16,24^, feature occlusion^25^, or surrogate models^18^. Concerning the evaluation of AMs, on the highest level, they can be either evaluated with respect to how well humans can understand the explanations^26,27^, or if the AMs are functioning correctly^28^.

Multiple properties of an AM can be examined to determine if it functions correctly, such as: *Robustness*^29–31^—an AMs sensitivity to input perturbations; *Complexity*^32,33^—the conciseness of explanations; or *Faithfulness*^2^—how accurately AMs identify prediction relevant features in the input. In this paper we investigate the faithfulness of AMs for neural time series classification models, given that we firmly believe that an AM should first and foremost faithfully reflect what is important to a model. Considering that there is no real consensus regarding the best approach for evaluating the faithfulness of AMs, multiple approaches have been proposed, which can be categorized as follows: *Sanity checks*^34,35^ define criteria that an AM has to fulfill (e.g., an AMs sensitivity to the model weights); ii) *localization-based approaches*^9,22^ compare the explanations with a human annotated ground-truth; iii) *re-training-based approaches*^36^ evaluate the accuracy change of a re-trained model, after removing the most relevant features according to an AM; and iv) *perturbation-based approaches*^2,3,16,22,25^ measure the effect of perturbing the most relevant features on a model’s output.

Looking into recent surveys on XAI and AMs, it is evident that most AMs have been created or evaluated with a focus on image and text data^37–39^, and highlight the lack of literature concerning AMs for neural time series models^40^. Fortunately, a growing body of work on AMs for time series is emerging. Sood et al.^41^ proposed a permutation-based and model-agnostic approach for estimating global feature importance in temporal models, while Bento et al.^42^ adapted KernelSHAP for RNNs. Moreover, Tonekaboni et al.^43^ introduced an AM which generates attributions for single time series.

To determine the faithfulness of AMs we apply an adapted methodology derived from region perturbation^3^, the most popular approach in literature. Region perturbation modifies the input features according to their relevance as estimated by an AM, either starting from the Most Relevant First (*MoRF*) or Least Relevant First (*LeRF*). The input features are modified in fixed sized regions, by using a user-defined *Perturbation Method (PM)*. Iteratively, the regions of the input are perturbed, and after each perturbation the change in the model’s prediction is recorded. Plotting the model predictions after each perturbation produces a *perturbation curve*. Perturbing the most relevant features should cause the model prediction to deteriorate quickly, and produce a small *Area Under the Perturbation Curve* ($AUPC_{MoRF}$) as opposed to perturbing the least relevant features, which should produce a large *Area Under the Perturbation Curve* ($AUPC_{LeRF}$).

Removing only the aspects from the input which were important for the model to make its predictions is not a trivial task, given that by using a wrong PM a shift in the data distribution may be caused^36^. This can cause both MoRF and LeRF perturbation curves to degrade (almost) equally quickly. Consequently, by using solely the $AUPC_{MoRF}$, it cannot be determined if the drop in the model’s class prediction output was caused by removing genuinely relevant features, or by a shift in the data distribution.

This is a common problem, as many other AM faithfulness evaluation approaches observe the effect of perturbing only the most important features, without taking into account how the PM selection may effect the evaluation^2,16,29,44,45^. Moreover, the value of either the $AUPC_{MoRF}$ or $AUPC_{LeRF}$ do not offer strong insights regarding an AMs faithfulness, without comparing the score to some baseline AM, such as by using a random feature relevance order.

An initial approach to mitigate this was proposed by Schulz et al.^46^ by introducing the *Degradation Score (DS)*. The Degradation Score is defined as the integral between the MoRF and LeRF perturbation curves, and quantifies to what extent an AM can separate high from low relevance features. However, a key limitation of the *DS* is that it does not consider *where* the differences between MoRF and LeRF perturbation curves occur. Our previously introduced Decaying Degradation Score (*DDS*) metric—which we also utilize in our extended experiments—addresses this by assigning greater weight to discrepancies involving the most and least important features, as early differences are more indicative of correct feature relevance ordering.

Schlegel et al.^47^ performed an initial perturbation-based faithfulness evaluation of AMs on time series data. They compared five AMs, with four PMs on a CNN and LSTM classifier trained for univariate time series. However, they did not examine the influence of the region size. In their methodology, they used a single deviation in the model’s output after perturbing a fixed percentage of the most relevant features. While this approach is computationally more efficient compared to sequential perturbations of individual regions, it may miss fluctuations in prediction scores. Such fluctuations can signal the use of an unsuitable PM or an inaccurate relevance ranking of features, both of which may compromise the reliability of the AM faithfulness estimation.

In a follow-up study, Schlegel et al.^48^ demonstrated that perturbation-based AM faithfulness evaluation is an effective method for assessing the faithfulness of AMs in time series data. In their experiments, they employed two different CNN architectures: one for two similar datasets and another for a distinct dataset. Rather than applying a single perturbation, they iteratively modified features whose relevance exceeded a certain threshold (e.g., the 90th percentile), gradually lowering this threshold with each step. They recorded the number of steps required for the model’s prediction to change from the original class to a different one. The study evaluated 16 PMs: six from their earlier work, two from ours, and eight new extended PMs. Additionally, to quantify the extent of the perturbations, they used both Euclidean distance and cosine similarity between the original and perturbed time series.

In their approach they relied only on the most relevant features, which has the previously mentioned limitations concerning the potential of using an unsuitable PM and causing a distribution shift. Additionally, while model complexity is not central to faithfulness evaluation, the CNNs used were relatively simple (three convolutional layers) compared to state-of-the-art models, and overfitted to the training data. They used a single model per dataset. Importantly, they found that the choice of PM significantly affects the outcomes of AM faithfulness evaluations. As a result, they recommend aggregating results across multiple PMs in future studies to ensure more robust conclusions.

Following, Schlegel et al.^49^ introduced the Attribution Stability Indicator, a metric that combines the various criteria explored in their previous work^48^, that according to them should be fulfilled after perturbation analysis is performed on an AM. Specifically, after perturbing a time series, the predicted class should change, prediction probability drop significantly, the explanations should differ strongly, and the original and perturbed time series should be similar. In their experiments they used a single PM based on previous results^48^, two CNN architectures for each of the investigated three datasets and six AMs.

However, this approach does not consider how choosing the wrong PM can severely affect the outcome of the evaluation. Neither does it take into account low relevance features. Moreover, in their methodology again a fixed percentage of the time series is perturbed at once, leading to the same issues as previously mentioned.

Using synthetic time series datasets, Ismail et al.^50^ compared AMs across different DL architectures, specifically, a CNN, Transformer and two LSTM variants. To estimate the faithfulness of AMs, they used precision and recall, to determine how many of the features that were marked as relevant were actually relevant compared to the ground-truth defined by the synthetic data generation process. However, a well-trained or complex model may actually only need a fraction of the features in the ground-truth to make a correct prediction, and an AM cannot be blamed for faithfully identifying the correct set of features on which the model relies. Therefore, we believe that relying on precision and recall can be misleading when evaluating the faithfulness of AMs. Additionally, they also evaluated AMs by applying a perturbation-based approach in which they replaced the most relevant features in steps of 10% of the input length. As a PM they used values from the original data distribution, given that the data generation process was known. However, this approach is not applicable on real-world data, since the data generation process most often cannot be replicated.

Turbé et al.^51,52^ developed a synthetic dataset featuring known discriminative attributes and adjustable complexity to support AM faithfulness validation on time series data. They evaluated the faithfulness of six AMs using a perturbation-based approach across three datasets—the synthetic dataset and two public datasets—as well as three model architectures (CNN, Bi-LSTM and Transformer). They introduced two new metrics for AM faithfulness estimation. The *AUCS*, a variant of the $AUPC_{MoRF}$, measures the impact of perturbing the most important features. The other metric, the *F*1*S*, uses the harmonic mean to quantify the difference of perturbing the most and least important features, although without accounting for the actual ranking of the most and least important features. Their study utilized a single PM, where random values are drawn from a normal distribution. To account for data distribution shifts, they injected the same noise in the training data as a form of data augmentation. However, this approach assumes retraining is feasible, which is an unrealistic scenario when selecting the most faithful AM for an available, already trained model. Moreover, as also indicated by Schlegel et al.^48^, other PMs may be more adequate for the AM evaluation.

Wei et al.^53^ extend the experiments of Turbé et al.^52^ using the same models, AMs, PM and faithfulness metrics. They introduced additional public datasets and new metrics to assess the robustness of AMs. These metrics quantify the robustness of AMs by measuring the kurtosis and skewness of the distribution of faithfulness scores across a representative dataset. In this context, negative skewness and higher kurtosis indicate more robust AMs, as they reflect consistent sharp prediction score drops across samples. While these metrics provide valuable insights into AM robustness, they may yield misleading results if the influence of different PMs is not accounted for.

Compared to previous evaluations of AM faithfulness for neural time series classification models, our work presents the most comprehensive experimental setup to date. We investigate a broader range of AMs, PMs and model architectures and explicitly consider the effect of region size selection, which is often an overlooked factor in earlier studies. Our study offers several key advancements. Our proposed *CMI* metric combines the *DDS*, which utilizes high-relevance and low-relevance features and emphasizes early differences between most and least relevant features, and *PES* which quantifies the consistency of AM faithfulness across the dataset. Additionally, our methodology employs *multiple* PMs weighted according to their suitability for the specific dataset and model. While this is computationally more expensive, it yields a more reliable assessment of AM faithfulness. To reduce overfitting and increase robustness, we train multiple models per architecture for each dataset, ensuring that our findings are not model-specific. Finally, we conduct an in-depth investigation of how PM selection and AM faithfulness vary across different dataset characteristics and model architectures, while also considering the impact of region size. These improvements directly address the limitations of prior methodologies and studies and significantly enhance the reliability of AM faithfulness evaluations.

Lastly, given the missing consensus which methodology should be used to estimate AM faithfulness, some authors argue black-box models in combination with AMs should generally be avoided^54^. Nevertheless, many other authors advocate for AMs^14,55,56^, since they open up the possibility for using DL models in high-stakes domains. The main reason being the recent advancements of DL models and the fact that DL models outperform interpretable models in accuracy and inference speed. Moreover, some authors argue that a whole understanding of a model may even be unnecessary, and a plausible explanation of a model’s prediction is enough^55^.

Due to the aforementioned reasons, it is evident that improvements in AM validation methodologies would be highly beneficial, as it would open up the path for the use of highly accurate black-box models in high-stakes domains.

## Problem statement

Given a univariate time series dataset *D*, where all time series are of equal length *N*, and each sample $x = [t_1,\ldots, t_N]$ consists of values $t_i$ at time step *i*. A model *M* is trained to classify samples $x \in D$. An attribution method *a* is a function which given a model *M* and a sample *x* produces a relevance vector $r = [r_1,\ldots, r_N]$ containing a relevance score for each $t_i$ in *x*. The individual relevance scores $r_i$ estimate how much each time step $t_i$ of *x* influenced the class score output of *M*.

Let *A* be a collection of AMs, from which we want to identify which $a \in A$ provides the most faithful explanations. To achieve this, region perturbation is employed which consecutively modifies a fixed number of input features defined by a region size $r_s$, using a perturbation method *p*. From an extensive set of perturbation methods *P*, we also aim to determine which $p \in P$ are most and least suitable for a given *D* and *M*.

A suitable PM for region perturbation induces *substantial change* in a specific class score output of *M* when modifying genuinely *relevant features*, and induces *little to no change* in the class score output of *M* when modifying genuinely *irrelevant features*. Moreover, a suitable PM does so consistently over all $x \in D$.

Finally, we aim to understand the impact of $r_s$ on the suitability of $p \in P$ depending on the properties of *D* and *M*.

## Methods for faithfulness estimation

In this section, we will first introduce the faithfulness metrics related to our experiments. In the second part, the adapted AM faithfulness evaluation methodology is presented.

### Metrics

We will first reiterate on the reasoning from our previous paper^13^ on why $AUPC_{MoRF}$ should not be used by itself for faithfulness evaluation of AMs. Following, we will reintroduce the Decaying Degradation Score (*DDS*) and Perturbation Effect Size (*PES*), and highlight how they address the issues of the $AUPC_{MoRF}$ metric. Afterwards, we introduce the novel Consistency-Magnitude-Index (*CMI*), a combination of the *DDS* and *PES*, and demonstrate its usage on a toy example.

#### Problems of $AUPC_{MoRF}$

As described in “Background” section, $AUPC_{MoRF}$ is the area under the perturbation curve after a fixed percentage of the most relevant input features have been perturbed. However, by relying only on the most relevant features, the AM faithfulness evaluation can yield results which may lead to wrong conclusions. Here we list the most prominent issues that we observed.

**Fig. 2:**
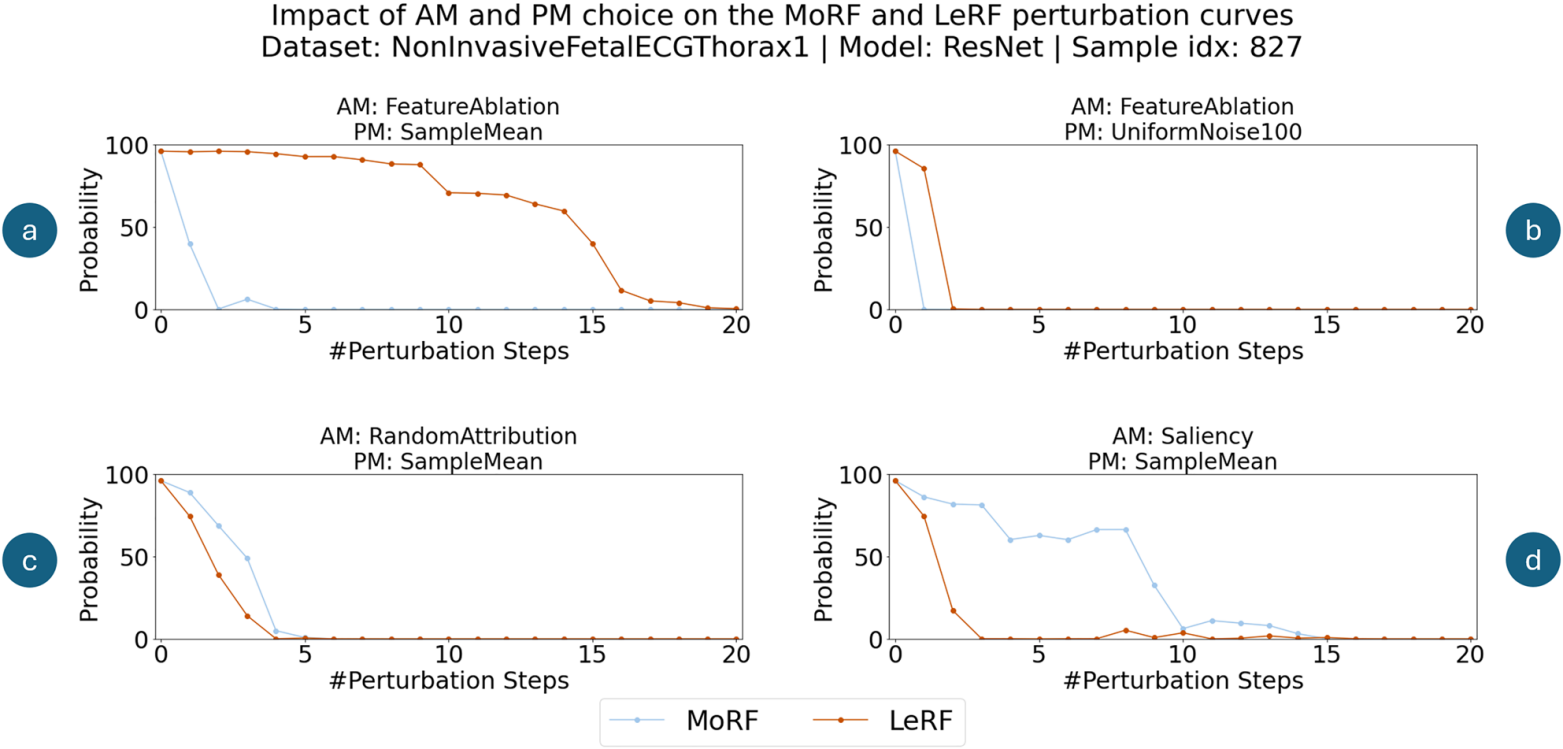
Impact of AM and PM selection on the MoRF and LeRF perturbation curves for the same sample, model and region size: (**a**) AM identifies relevant and irrelevant features correctly—PM removes only prediction relevant information; (**b**) AM identifies relevant and irrelevant features correctly—perturbation of irrelevant features strongly impacts model prediction; (**c**) AM does not discern relevant from irrelevant features; (**d**) AM reverses feature relevance.

*[Issue 1] Effects of bad PM choice not evident:* Figure 2a and b show two exemplary MoRF and LeRF perturbation curve pairs using the same sample, model and explanation, however different PMs have been used to evaluate the faithfulness of the explanation. By considering *only* the MoRF perturbation curves (blue lines), one would deduce that the PM used in Fig. 2 b is superior, given that the probability of the predicted class dropped to 0 after a single perturbation. However, by considering also the LeRF perturbation curves (red lines), it is evident that the PM in Fig. 2b modified the input too strongly, and actually pushed the sample out of distribution. This caused the classifier to reduce the probability from 1 to 0 of the predicted class, after 2 perturbations independently of the features actual importance.

*[Issue 2] Bad AM not apparent without comparison to a baseline AM:* Typically, when the $AUPC_{MoRF}$ metric is used for faithfulness evaluation of AMs, a known *bad* baseline AM has to be used to compare the evaluated AMs. Generally, this baseline AM perturbs features in a random order, e.g., by assigning random relevances sampled from a uniform distribution to each feature. Figure 2c depicts the MoRF and LeRF perturbation curves of a sample, for which an explanation of random relevances was evaluated. As it can be seen, the MoRF perturbation curve may decrease slowly, and even appear plausible, when considered in isolation. However, by including also the LeRF curve, it is evident that the explanation method cannot discern relevant from irrelevant features, given that they decrease in a similar fashion. Therefore, by relying only on the $AUPC_{MoRF}$ we would need another AM to compare against. It would be desirable, that a faithfulness metric reflects that the outcome of the evaluation is bad, without the necessity of a comparison AM.

*[Issue 3] Relevance reversal not noticeable:* Focusing solely on the $AUPC_{MoRF}$ can lead to the misleading conclusion that certain AMs perform worse than random. This limitation is because the $AUPC_{MoRF}$ cannot detect when an AM systematically reverses feature relevance, offering little insight into low scores and leaving the root cause unclear. If systematic reversals are detectable, AMs can still be valuable, as their explanations can be corrected without modifying the method itself. Figure 2d shows such an example, where the MoRF perturbation curve slowly degrades, while the LeRF perturbation curve declines rapidly. When only a limited selection of AMs is compatible with a chosen model, metrics capable of detecting feature relevance reversal become especially valuable.

*[Issue 4] No information regarding how consistently an AM works well:* A major problem of $AUPC_{MoRF}$ is that it does not provide any information on how consistently an AM is able to separate relevant from irrelevant features. $AUPC_{MoRF}$ is reported as the average over a set of samples (e.g., test set). However, a relatively high $AUPC_{MoRF}$ may be achieved if the case that an AM is able to separate relevant from irrelevant features very well only for a subset of the samples (e.g., specific class). While averaging is a common issue in performance evaluation across domains, including model performance, in high-stakes or unbalanced settings, relying solely on averages is often avoided. Similarly, reporting only the average $AUPC_{MoRF}$ can hide inconsistent AM behavior. Thus, a metric that captures consistency across all samples is vital for accurately evaluating an AMs faithfulness.

#### Decaying Degradation Score (*DDS*)

The Decaying Degradation Score (*DDS*) is a metric which captures the extent to which an AM is able to separate relevant from irrelevant features. It is defined as the sum of weighted differences between the MoRF and LeRF perturbation curves at each perturbation step. It uses a cubic weighting scheme, which adds more weight to the differences of the first perturbation steps. This produces a higher score if the actually most and least important features are assigned the highest and lowest relevances. The *DDS* is defined as follows:

1 $$\begin{aligned} DDS = \sum \limits _{i=1}^{n} \left( (L_i - M_i) \cdot \left( \frac{n-i+1}{n}\right) ^3 \right) \end{aligned}$$

In addition to the *DDS* introduced in our previous work, we normalize it by dividing it with the maximum possible *DDS* value for the evaluated samples, which solely depends on the number of performed perturbation steps. The maximum DDS can only be achieved if there is only a single feature which causes the classifier’s predicted class probability to change from 1 to 0 after a single perturbation, and perturbing other features does not impact the classifier prediction anymore. The maximum achievable *DDS* value is calculated as follows:

2 $$\begin{aligned} DDS_{max} = \sum \limits _{i=2}^{n} \left( \frac{n-i+1}{n}\right) ^3 \end{aligned}$$

After normalization, the *DDS* will be a real value in the range of $[-1,1]$. A value of 0 implies that there is no difference between the MoRF and LeRF perturbation curves, while 1 means that only a single feature was important for the prediction and it was correctly identified as the most important one. Negative *DDS* values imply that the AM reverses feature relevances. However, it is important to note that in most cases there are several features that are important for a prediction, and therefore *DDS* of 1 is fairly rare.

By relying on the *DDS* it is evident how Issues 1, 2 and 3 of $AUPC_{MoRF}$ can be addressed. In the case that a bad PM has been selected (Issue 1), which causes the classifier’s output to change from 1 to 0 independently of the features actual relevance, the *DDS* will be 0 or close to 0, since both the MoRF and LeRF perturbation curves are very similar. Also, by using the *DDS* we do not need a *bad* baseline AM anymore (Issue 2), since the score by itself has meaning. If the *DDS* is very close to 0 for an AM, we know it should be avoided. Finally, if the AM systematically mistakes relevant and irrelevant features (Issue 3), the *DDS* will become negative.

#### Perturbation Effect Size (*PES*)

The Perturbation Effect Size (*PES*) is a measure, which quantifies how consistently an AM is able to separate relevant from irrelevant features over a set of samples. In our previous paper, we defined the *Perturbation Effect Size* (*PES*) using Kerby’s simple difference formula^57^ with the hypothesis “*The* $AUPC_{MoRF}$ *values are lower than the* $AUPC_{LeRF}$  *values*”. This hypothesis considers only the simple difference between the perturbation curves, as was used by Schulz et al.^46^ in their Degradation Score metric. The equivalent hypothesis for the *PES* using the Degradation Score could also be re-stated as *“The Degradation Score is positive”*, since the Degradation Score is just the difference between the $AUPC_{MoRF}$ and $AUPC_{LeRF}$.

Given that we want to utilize the benefits of the weighted differences of the perturbation curves as is used in the *DDS* also for the *PES*, in this work we updated the hypothesis. Therefore, the hypothesis for the updated *PES* is *“The Decaying Degradation Score is positive”* and thus the *PES* formula becomes:

3 $$\begin{aligned} f= & \frac{\# ({{DDS}} > 0)}{\# {{samples}}} \end{aligned}$$

4 $$\begin{aligned} u= & \frac{\# ({{DDS}} < 0)}{\# {{samples}}} \end{aligned}$$

5 $$\begin{aligned} PES= & f - u \end{aligned}$$

The *PES* produces a real value in the range of $[-1,1]$, which measures the relation of how often relevant from irrelevant features were separated correctly over a set of samples. If $PES = 0$, the *DDS* values are equally often positive and negative. Consequently, a PM causing such a *PES* would be deemed unsuitable. An ideal PM would produce $PES = 1$, meaning that the *DDS* is always positive over the set of evaluated samples, or alternatively $PES = -1$ in the case that an AM consistently reverses relevant and irrelevant features.

Evidently, by utilizing the *PES* metric, it becomes simple to estimate how consistently an AM separates relevant from irrelevant features over a set of samples, addressing Issue 4 of $AUPC_{MoRF}$.

#### Consistency-Magnitude-Index (*CMI*)

To streamline the identification of the most faithful AM, it is desirable to use only a single faithfulness metric. Therefore, to exploit the benefits of both metrics, we combined the *DDS* and *PES* into a single new metric, the *Consistency-Magnitude-Index* (*CMI*).

In our previous paper we stated that the *DDS* and *PES* are complimentary measures and that it is best if they are *in agreement* and *high*. If either one of the metrics is low, it should be penalized in the combined metric. For example, this can occur when a PM modifies the input only slightly, causing a very small positive *DDS* over most samples, yielding in a relatively high *PES*. An established method of combining two metrics into a single score, while penalizing strong disagreement is the *harmonic mean* (e.g., used in F1-score).

However, the harmonic mean is not defined for negative values, while both the normalized *DDS* and *PES* are in the range of $[-1,1]$. To adopt the harmonic mean for combining *DDS* and *PES*, we have to consider three possible cases to account for the signs of the *DDS* and *PES*: (i) *DDS* and *PES* are both positive, (ii) *DDS* and *PES* are both negative, (iii) *DDS* and *PES* conflicting—one positive, other negative.

By taking the absolute value of the *DDS* and *PES* if they are both either positive or negative, we can assure that we use values for which the harmonic mean is defined. As a reminder, high negative *DDS* and *PES* values mean that the AM is still able to distinguish relevant from irrelevant features, only the relevance order is reversed.

This leaves the case where *DDS* and *PES* have conflicting signs. This case occurs mostly when either the PM modifies the input insufficiently, or when the AM is not able to distinguish relevant from irrelevant features, causing the *DDS* and *PES* to be close to 0, but with opposite signs. Since an AM that obtains conflicting *DDS* and *PES* is not desirable, we assign 0 to the *CMI* in this case. Therefore, the *CMI* is defined as follows:

6 $$\begin{aligned} CMI = {\left\{ \begin{array}{ll} \frac{2}{ |DDS|^{-1} + |PES|^{-1}} & \text {if } DDS \cdot PES \ge 0\\ 0 & \text {otherwise} \end{array}\right. } \end{aligned}$$

Given that we use the absolute values of *DDS* and *PES*, the *CMI* is in the range of [0, 1], where a higher value indicates a more faithful AM. By using a single metric, which incorporates both the benefits of the *DDS* and *PES*, it is easy to rank, and therefore identify the most faithful AMs.

### Methodology for AM faithfulness estimation

As stated in Section “Problem statement”, the main goal is, from a set of AMs, to identify the AM which provides the most faithful explanations for a given dataset and model. In our previous work^13^, we presented the strong impact PM selection can have on the faithfulness evaluation. However, a major issue is that the PM (and region size) have to be selected *before* the evaluation can be performed. However, a PMs suitability can only be assessed *after* the evaluation has been performed. As we will show later in the experimental results (Section “Results”), a PMs suitability is affected by both dataset and model properties, and can even depend on the sample class.

**Fig. 3:**
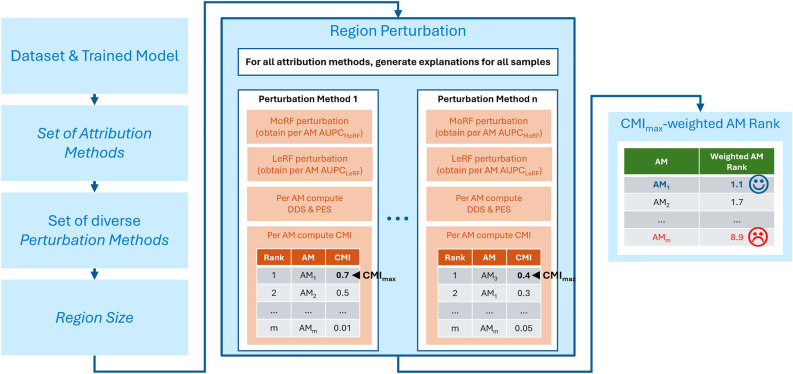
Overview of the adapted methodology for evaluating AM faithfulness: Given a dataset and model, a set of AMs, PMs and region size has to be selected. Region perturbation is then applied with each PM to compute a *CMI*-based AM ranking per PM. The final rank of each AM is is determined by computing the weighted average across all rankings, where the weight is the squared highest achieved *CMI* value ($CMI_{max}$) per ranking.

Therefore, we propose an adapted methodology for evaluating the faithfulness of AMs that takes into account a PMs suitability for the used dataset and model, depicted in Fig. 3.

Given a dataset and trained model, first it is necessary to select a set of AMs from which the most faithful one should be identified. The set of investigated AMs is influenced by the availability of existing implementations of AMs compatible with the used model, as well as their performance and access to the model.

Subsequently, a *set of diverse PMs* is selected that should be used with region perturbation. By utilizing a set of diverse PMs the risk of selecting a bad PM beforehand can be drastically reduced. Although, it has to be kept in mind that increasing the number of PMs prolongs the evaluation linearly.

In addition to the set of PMs, a fixed region size has to be selected, which according to the results of our previous paper should neither be too large, nor too small (e.g., 2–5% of input length).

Next, region perturbation can be performed. With each AM, explanations for all samples of the dataset are computed. Thereafter, using each PM, the *CMI*-based ranking of AMs is obtained. To do this, region perturbation is performed for every AM on each sample of the dataset with the obtained explanations in MoRF and LeRF order. Using the MoRF and LeRF perturbation curves the normalized *DDS* of each sample can be computed, and therefore the mean *DDS* and *PES* as well. The mean *DDS* and *PES* are then used to obtain the *CMI* for each AM. Subsequently, a ranking of the AMs is gained by ordering the AMs according to their *CMI* score in descending order. This process is repeated with each PM, to obtain a set of AM rankings. Different PMs may yield different rankings of AMs. However, the CMI of the best performing AM will indicate the suitability of the PM for the faithfulness evaluation.

To obtain the most faithful AM, the $CMI_{max}$-*weighted average of the AM ranks* of each AM given a PM is taken. The weight, with which the AM rank is multiplied is the squared highest achieved CMI value with a given PM, which we will refer to as $CMI_{max}$. With this, it is ensured that rankings of suitable PMs are taken more into account than rankings of PMs which were unsuitable. The most faithful AM then, from the set of AMs is the one with the best average weighted rank. To get a better understanding of the process, let’s look at a toy example.

*Toy example:* In this toy example we are provided with a dataset and model trained on it. Our goal is to identify the most faithful AM between the following four: IntegratedGradients, GuidedGradCAM, FeatureAblation and DeepLIFT. To do so, we will apply the above describe methodology using two substantially different PMs: UniformNoise—replace the features with values sampled from a uniform distribution; and SampleMean—replace the features with the samples mean. As region size, we use 2.5% of the input length.

Therefore, we first compute the explanations of all samples of the dataset for the given model. Then, with each PM, we perturb the samples according to the explanations in the MoRF and LeRF order to obtain the per sample $AUPC_{MoRF}$ and $AUPC_{LeRF}$ values. With all the $AUPC_{MoRF}$ and $AUPC_{LeRF}$ we can compute the per dataset *DDS* and *PES* and in turn obtain the per AM *CMI* with each PM. The *CMI* ranking of AMs for the UniformNoise PM can be seen in Table 1, and for SampleMean in Table 2.

**Table 1:** Toy Example—ranking of AMs using UniformNoise.

Rank	AM	CMI
1	FeatureAblation	0.0491
2	IntegratedGradients	0.0450
3	GuidedGradCAM	0.0412
4	DeepLIFT	0.0251

**Table 2:** Toy Example—ranking of AMs using SampleMean.

Rank	AM	CMI
1	IntegratedGradients	0.4506
2	GuidedGradCAM	0.4247
3	FeatureAblation	0.3099
4	DeepLIFT	0.1605

In this toy example, it can be seen that the two different PMs generate two substantially different rankings, and that the *CMI* for the best AM is vastly different. Therefore, to obtain a conclusive ranking, the AM ranks are averaged across PMs, while taking into account how *good* a PM actually was. To quantify how good the PM actually was, the highest achieved *CMI* ($CMI_{max}$) over all AMs is used. Finally, for each AM we compute the $CMI_{max}$ weighted average AM rank using the following formula:

7 $$\begin{aligned} Rank_{AM} = \frac{\sum \nolimits _{i=1}^{n}w_iX_i}{\sum \nolimits _{i=1}^{n}w_i} \end{aligned}$$

Where $w_i$ is the squared $CMI_{max}$ per PM, and $X_i$ the rank of the AM for this PM. As it can be seen from Table 1$w = 0.0491^2 \approx 0.0024$ for UniformNoise, and from Table 2$w=0.4506^2 \approx 0.203$ for SampleMean. Subsequently, the weighted average rank for each AM can be calculated as follows:

8 $$\begin{aligned} Rank_{IntegratedGradients}= & \frac{ (2 * 0.0024) + (1 * 0.203) }{ 0.0024 + 0.203 } = \frac{0.2078}{0.2054} \approx {\textbf {1.017}} \end{aligned}$$

9 $$\begin{aligned} Rank_{GuidedGradCAM}= & \frac{ (3 * 0.0024) + (2 * 0.203) }{ 0.0024 + 0.203 } = \frac{0.4132}{0.2054} \approx 2.017 \end{aligned}$$

10 $$\begin{aligned} Rank_{FeatureAblation}= & \frac{ (1 * 0.0024) + (3 * 0.203) }{ 0.0024 + 0.203 } = \frac{0.6114}{0.2054} \approx 2.977 \end{aligned}$$

11 $$\begin{aligned} Rank_{DeepLIFT}= & \frac{ (4 * 0.0024) + (4 * 0.203) }{ 0.0024 + 0.203 } = \frac{0.8216}{0.2054} = 4 \end{aligned}$$

It can be seen that in this case IntegratedGradients provides the most faithful explanations. It can also be seen, that the ranking provided by the UniformNoise PM was taken significantly less into account, due to the lower suitability of this PM for the provided dataset and model. Even though this is a just a toy example, it can be seen how by using the $CMI_{max}$ weighted average AM rank, the effects of bad PMs can be mitigated, and how it can be extended to include additional AMs, and especially more diverse PMs.

## Experiments

Compared to our previous paper, we extended the experiments by including additional AMs (+3), additional PMs (+15)—including time series specific PMs, and additional model architectures (+3). Table 3 provides an overview of the investigated datasets, model architectures, AM, PMs and region sizes. Through the extended experiments we aim to demonstrate the advantages of our adapted AM faithfulness evaluation methodology, take a closer look at the differences between dataset types and model architectures, and find answers to questions with respect to:

- Perturbation methods:
  - Which PMs are generally suitable across different dataset types for evaluating AMs
  - Which PMs should be avoided?
  - How important is PM selection for specific dataset types and model architectures in region perturbation?
- Attribution methods:
  - Are there AMs that work generally well across all the investigated datasets and model architectures?
  - Which AMs should generally be avoided?
  - How important is AM selection for specific dataset types and model architectures in region perturbation?
- Region size:
  - What is the influence of region size on AM faithfulness evaluation
  - Is a smaller region size preferable for all investigated datasets and model architectures?
  - Do certain PMs work better with a smaller or larger region sizes considering the different dataset types?
  - Do certain AMs work better with a smaller or larger region sizes considering the different dataset types?

**Table 3:** Overview of the investigated datasets, model architectures, AMs PMs and region sizes. Italicized entries are new compared to our previous work.

Datasets	FordA, FordB, NonInvasiveFetalECGThorax1 (ECG1), NonInvasiveFetalECGThorax2 (ECG2), Wafer
Model architectures	ResNet, Inception, *MLP*, *LSTM*, *ViT*
Attribution methods	GradCAM, Saliency, Integrated Gradients, DeepLIFT, InputXGradient, GuidedGradCAM, KernelSHAP, LIME, RandomAttribution, *Feature Ablation*, *Deconvolution*, *Guided Backprop*
Perturbation methods	SampleMean, Zero, SubsequenceMean, OutOfDistHigh, OutOfDistLow, Inverse, Swap, UniformNoise100, *UniformNoise50*, *UniformNoise25* *Padding*, *Laplace*, *GaussianBlur*, *SavitzkyGolay*, *LinearInterpolation*, *QuadraticInterpolation*, *CubicInterpolation*, *Nearest*, *LeftNeighborWindow*, *RightNeighborWindow*, *NearestNeighborWindow*, *SimilarNeighborWindow*, *DissimilarNeighborWindow*
Region sizes	2.5% and 10% of input length

### Experimental setup

*Dataset selection:* The same 5 univariate time series datasets from the UCR time series archive^58^ were used as in our previous paper, namely: *FordA*, *FordB*, *NonInvasiveFetalECGThorax1*, *NonInvasiveFetalECGThorax2* & *Wafer*. These datasets can be categorized into the following three groups: i) balanced binary classification datasets (FordA, FordB); ii) multiclass classification datasets (NonInvasiveFetalECGThorax1, NonInvasiveFetalECGThorax2); and iii) imbalanced binary classification—anomaly detection (Wafer). We selected these datasets primarily due to the high number of training samples that they had (to reduce overfitting probability), and due to the fact that a high accuracy was achievable (to ensure model learned the class specific characteristics). In our experiments we focused primarily on univariate time series to reduce the number of input features that have to be processed, due to the already high complexity of the experiments. In the “Practical example: AM faithfulness evaluation for a multivariate time series classifier” section we demonstrate the proposed methodology on multivariate time series data as well.

*Model architectures:* Five model architectures were used in our experiments, for which multiple models were trained per dataset. Two of the model architectures that were also present in our previous paper are based on the best-performing state-of-the-art DL architectures for time series classification according to Fawaz et al.^7,8^, namely ResNet and InceptionTime. These model architectures are CNNs, and were selected due to their good performance for time series classification. Additionally, we included a multilayer perceptron (MLP)^59^, due to its architectural simplicity. We also included an LSTM^60^, due to the fact that this architecture was created for sequential data. Finally, given that computer vision model architectures show promising results for time series classification, we additionally included a vision transformer (ViT)^61^, that we adapted for time series classification. A description of the exact model architectures is provided in the Appendix.

*Model training:* All datasets provided a distinct training and test set. For the validation set, 20% of the training set samples were selected using stratified sampling. The models were trained for up to 500 epochs, with a batch size of 256 and Adam as the optimizer with the default settings. For each model architecture and dataset combination, 30 models with different initial seeds were trained. The Tree-structured Parzen Estimator^62^ was used to set the learning rate in the range of $[1e-6,1e-2]$. As many as 30 different learning rates were tried for each model. To reduce model training time, a model was saved as soon as the validation accuracy passed a threshold close to the accuracies of the state-of-the-art models (FordA $\ge 93\%$, FordB $\ge 92\%$, NonInvasiveECGFetalThorax1 $\ge 93\%$, NonInvasiveECGFetalThorax2 $\ge 93\%$, Wafer $\ge 99\%$). In the case that a model never passed the threshold, the most accurate model was saved. Following, for each model architecture, five models with the highest F1-score on the test set were selected for detailed examination.

*Attribution methods:* We evaluated the faitfhulness of the following AMs: GradCAM^22^, Saliency^20^, Integrated Gradients^21^, DeepLIFT^24^, InputXGradient^23^, GuidedGradCAM^22^, KernelSHAP^15^, LIME^18^, Feature Ablation*^25^, Deconvolution*^25^, Guided Backprop*^63^. The AMs marked with an asterisk were not included in our previous paper. Moreover, as the baseline for a bad AM, a random relevance approach was used (RandomAttribution), where the relevance of each feature was defined by a value sampled from a uniform distribution in the range [0, 1). The investigated AMs were provided by Captum^64^ with their default settings, except for GradCAM and RandomAttribution, which were implemented by ourselves. Moreover, GradCAM and GuidedGradCAM were only used with the ResNet and Inception model architectures, since these AMs only work for CNNs. Using all investigated AMs, the explanations for all samples of the provided test sets were computed with each model.

*Perturbation methods:* In addition to the 8 PMs investigated in our previous paper, we included 15 additional PMs, which are related to noise injection, smoothing and imputation of time series data. This brings the total number of investigated PMs to 23. We categorize the investigated PMs into the following categories: baseline (2), constants (4), noise injection (5), smoothing (3), interpolation (9). *Baseline* PMs are the baseline of what we consider bad PMs, and aim to set the values of the perturbed features out of distribution. *Information removal* PMs try to replace the features with a non-informative constant value, e.g., 0. *Noise injection* PMs add noise to the time series, while *smoothing* PMs remove noise by smoothing the time series. Finally, *interpolation* PMs treat the features that should be perturbed as if they were missing, and fill in the region by means of interpolation. The definition and categorization of all investigated PMs are provided in Tables 4 and 5.

*Faithfulness evaluation methodology:* To evaluate the AMs with the previously defined PMs, we used the methodology outlined in the “Methodology for AM faithfulness estimation” section. Each sample was perturbed with every PM in the MoRF and LeRF order with a smaller (2.5% input length), and a larger (10% input length) region size. We opted for relative instead of fixed region sizes given that it allows a comparison between datasets. While we previously showed that smaller region sizes were better, we also included a much larger region size to observe the effect it has on the suitability of PMs with different dataset types and model architectures. Each time series was split into subsequences (regions) of consecutive time points defined by the region size. The region relevance was calculated using the mean of the relevances inside of the subsequence. Perturbation was stopped after 50% of the input features had been modified. This assures that all features are perturbed, since time series are perturbed in both the MoRF and LeRF order. After perturbing all time series of a dataset, the dataset *DDS* and *PES* were computed for all combinations of model architecture, model, AM, PM and region size, which in turn was used for computing the *CMI* for AM ranking.

To summarize, we performed region perturbation on each sample from the test set of 5 datasets 27 600 times: 5 model architectures, 5 trained models per architecture (different initial seeds), 12 AMs, 23 PMs, 2 region sizes and 2 perturbation orders. The evaluation source code including the trained models is publicly available on GitHub under the following URL: https://github.com/perturbationeffect/cmi-am-validation-for-dl-ts-classifiers.

**Table 4:** Definition of investigated *baseline*, *constants*, *noise injection* and *smoothing* perturbation methods: perturbation methods applied on a time series sample $x = [t_1,\ldots, t_N]$ consisting of values $t_i$ sampled at time step *i*, where $x'$ is the modified time series and $t'_i$ the modified value at time step *i*.

PM	Category	Modification procedure
OutOfDistHigh	Baseline	$t'_i = a \cdot 100$, given $a = \max \{|\max t_i|, |\min t_i| \}$
OutOfDistLow	Baseline	$t'_i = -(a \cdot 100)$, given $a = \max \{|\max t_i|, |\min t_i| \}$
SampleMean	Constants	$t'_i = \frac{1}{N} \sum \limits _{j=1}^{N} t_j$
Zero	Constants	$t_i = 0$
SubsequenceMean	Constants	$t'_i = \frac{1}{k-j}\sum \limits _{l=j}^{k} t_l$, given $j,k \in \{1,\ldots,N\}$ and $j < k$
Padding	Constants	$[t_j,\ldots, t_k] = [t_{j-1},\ldots, t_{j-1}]$ given $j,k \in \{1,\ldots,N\}$ and $j < k$
UniformNoise100	Noise injection	$t_i \sim Unif(-1, 1)$
UniformNoise50	Noise injection	$t_i = (t_i \cdot 0.5) + (a \cdot 0.5)$, given $a \sim Unif(-1, 1)$
UniformNoise25	Noise injection	$t_i = (t_i \cdot 0.75) + (a \cdot 0.25)$, given $a \sim Unif(-1, 1)$
Inverse	Noise injection	$t'_i = \max \{t_1,\ldots, t_N\} - t_i$
Swap	Noise injection	$[t_j, t_{j+1},\ldots, t_k] = [t_k, t_{k-1},\ldots, t_j]$ given $j,k \in \{1,\ldots,N\}$ and $j < k$
Laplace	Noise injection	$x' = x \star g$, where $g = [1\; -2 \quad 1]$
GaussianBlur	Smoothing	$x' = \frac{1}{2\pi \sigma ^2}e^{-\frac{x^2}{2\sigma ^2}}$, where $\sigma =5$
SavitzkyGolay	Smoothing	$t'_i = \sum \limits _{j=\frac{1-m}{2}}^{\frac{m-1}{2}} C_j t_{i+j}$ where $m=21$ and $C_i$ are the coefficients of the 3rd degree polynomial

**Table 5:** Definition of investigated *interpolation* perturbation methods: perturbation methods applied on a time series sample $x = [t_1,\ldots, t_N]$ consisting of values $t_i$ sampled at time step *i*, where $x'$ is the modified time series and $t'_i$ the modified value at time step *i*.

Perturbation method	Category	Modification procedure
LinearInterpolation	Interpolation	$t'_i = t_j + (t_k - t_j) \cdot \frac{i-j}{k-j}$ where *j* is the index of the nearest preceding value to *i*, and *k* the nearest succeeding non-missing value
QuadraticInterpolation	Interpolation	$t'_i = t_j \left( \frac{(i-k)(i-l)}{(j-k)(j-l)}\right) + t_k \left( \frac{(i-j)(i-l)}{(k-j)(k-l)}\right) + t_l \left( \frac{(i-j)(i-k)}{(l-j)(l-k)}\right)$ where *j* and *k* are the indices of the nearest preceding values to *i*, and *l* the nearest succeeding non-missing value
CubicInterpolation	Interpolation	$t'_i = t_j \left( \frac{(i-k)(i-l)(i-m)}{(j-k)(j-l)(j-m)}\right) + t_k \left( \frac{(i-j)(i-l)(i-m)}{(k-j)(k-l)(k-m)}\right) + t_l \left( \frac{(i-j)(i-k)(i-m)}{(l-j)(l-k)(l-m)}\right) + t_m \left( \frac{(i-j)(i-k)(i-l)}{(m-j)(m-k)(m-l)}\right)$ where *j*, *k*, and *l* are the indices of the nearest preceding values to *i*, and *m* the nearest succeeding non-missing value
Nearest	Interpolation	$[t_j,\ldots, t_{j+((k-j)/2)}] = t_{j-1}$ $[ t_{j+((k-j)/2)+1},\ldots, t_{k}] = t_{k+1}$ given $j,k \in \{1,\ldots,N\}$ and $j < k$
LeftNeighborWindow	Interpolation	$${[}t_j,\ldots, t_k] = \left\{ \begin{array}{ll} [t_j,\ldots, t_k] & \text {if } j = 0 \\ {[}t_{j-(k-j)},\ldots, t_{j-1}] & \text {otherwise} \end{array} \right.$$ given $j,k \in \{1,\ldots,N\}$ and $j < k$
RightNeighborWindow	Interpolation	$$[t_j,\ldots, t_k] = \left\{ \begin{array}{ll} [t_j,\ldots, t_k] & \text {if } k = N\\ {[}t_{k+1},\ldots, t_{k+(k-j)}] & \text {otherwise} \end{array} \right.$$ given $j,k \in \{1,\ldots,N\}$ and $j < k$
NearestNeighborWindow	Interpolation	$$[t_j,\ldots, t_k] = \left\{ \begin{array}{ll} [t_j,\ldots, t_k] & \text {if } k = N \vee j = 0\\ {[}t_{s},\ldots,t_{j-1},t_{k+1},\ldots,t_{e}] & \text {otherwise} \end{array} \right.$$ given $j,k \in \{1,\ldots,N\}$ and $j < k$ where $s = j-(\frac{k-j}{2})$ and $e = k+(\frac{k-j}{2})$
SimilarNeighborWindow	Interpolation	$$[t_j,\ldots, t_k] = \left\{ \begin{array}{ll} LEFT & \text {if } k = N \vee DTW(LEFT,[t_j,\ldots, t_k]) < DTW(RIGHT,[t_j,\ldots, t_k])\\ RIGHT & \text {otherwise} \end{array} \right.$$ given $j,k \in \{1,\ldots,N\}$ and $j < k$ where $LEFT = [t_{j-(k-j)},\ldots, t_{j-1}]$ and $RIGHT = [t_{k+1},\ldots, t_{k+(k-j)}]$
DissimilarNeighborWindow	Interpolation	$$[t_j,\ldots, t_k] = \left\{ \begin{array}{ll} LEFT & \text {if } k = N \vee DTW(LEFT,[t_j,\ldots, t_k]) > DTW(RIGHT,[t_j,\ldots, t_k])\\ RIGHT & \text {otherwise} \end{array} \right.$$ given $j,k \in \{1,\ldots,N\}$ and $j < k$ where $LEFT = [t_{j-(k-j)},\ldots, t_{j-1}]$ and $RIGHT = [t_{k+1},\ldots, t_{k+(k-j)}]$

### Sanity check: AM faithfulness on untrained model

Before presenting and discussing the experimental results of the trained models, we first conduct a sanity check of the *CMI* metric. Specifically, we compute the introduced metric for an untrained model and compare it to its trained counterpart. Since an untrained model has not learned any meaningful patterns from the data, all generated explanations should essentially consist of noise. Consequently, there should be no clear distinction between relevant and irrelevant features, causing the *CMI* metric to remain consistently close to 0, regardless of the chosen AM or PM.

Table 6 compares the highest achieved *CMI* value per PM ($CMI_{max}$), across all investigated AMs of a trained and untrained ResNet model on the FordA dataset using a smaller region size. Here we can confirm that for an untrained model, the *CMI* metric correctly reflects the uncertainty of the model, as there is barely any distinction between relevant and irrelevant features, thus leading to scores of essentially 0. Contrary, for the trained model a clear distinction between PM suitability can be seen.

Additionally, examining the individual *CMI* scores of all AMs for the untrained model using any PM, reveals only insignificant differences, due to the extremely low scores. This is shown in Table 7, which presents the *CMI* scores of all AMs after applying the *Padding* and *OutOfDistLow* PMs, which had the highest $CMI_{max}$ for the trained and untrained model respectively.

Therefore, we can conclude that the proposed metric correctly reflects the poor feature relevance discrimination performance of an untrained model.

**Table 6:** Comparison of $CMI_{max}$ between a trained and untrained model—for the same dataset (FordA), model architecture (ResNet) and region size (2.5% input length). As expected, the untrained model achieves scores close to 0, since the model did not learn to distinguish relevant from irrelevant features.

Perturbation method	Trained	Untrained
Padding	0.4507	0.0008
LinearInterpolation	0.4313	0.0010
Nearest	0.4251	0.0008
SubsequenceMean	0.4061	0.0005
NearestNeighborWindow	0.4060	0.0013
Laplace	0.4045	0.0013
Swap	0.4022	0.0003
Zero	0.4008	0.0012
SampleMean	0.4008	0.0012
RightNeighborWindow	0.3912	0.0013
SimilarNeighborWindow	0.3912	0.0013
LeftNeighborWindow	0.3901	0.0014
DissimilarNeighborWindow	0.3883	0.0014
GaussianBlur	0.3771	0.0010
CubicInterpolation	0.2379	0.0004
UniformNoise50	0.2325	0.0012
UniformNoise75	0.2311	0.0012
UniformNoise100	0.2297	0.0012
UniformNoise25	0.2297	0.0012
QuadraticInterpolation	0.2274	0.0004
SavitzkyGolay	0.2169	0.0003
Inverse	0.1978	0.0008
OutOfDistLow	0.0000	0.0059
OutOfDistHigh	0.0000	0.0044

**Table 7:** Comparison of *CMI* per AM between a trained and untrained model—for the same dataset (FordA), model architecture (ResNet) and region size (2.5% input length). For the trained model a clear distinction between the *CMI* scores can be seen when applying the best performing PM *Padding*, while for the untrained model, there are only negligible differences both for the *Padding* and *OutOfDistLow* PM, that performed best on the untrained model.

Attribution method	Padding		OutOfDistLow	
	Trained	Untrained	Trained	Untrained
FeatureAblation	0.4507	0.0004	0.0	0.0059
DeepLIFT	0.3355	0.0008	0.0	0.0048
GradCAM	0.3114	0.0007	0.0	0.0059
IntegratedGradients	0.2221	0.0008	0.0	0.0047
InputXGradient	0.1283	0.0004	0.0	0.0024
Saliency	0.1111	0.0001	0.0	0.0002
GuidedGradCAM	0.0744	0.0000	0.0	0.0016
GuidedBackprop	0.0509	0.0000	0.0	0.0002
Deconvolution	0.0298	0.0001	0.0	0.0000
RandomAttribution	0.0093	0.0000	0.0	0.0001
KernelShap	0.0047	0.0001	0.0	0.0001
Lime	0.0000	0.0000	0.0	0.0003

### Results

In this section, we discuss the results of our experiments, focusing on the questions stated earlier in Section “Experiments”. First, we will analyze the performance of individual PMs, highlighting key findings regarding PM choice. Subsequently, we explore observations related to AM and region size. Following, we summarize our findings regarding dataset characteristics and model architectures. Due to the lengthy names of the *NonInvasiveFetalECGThorax1* and *NonInvasiveFetalECGThorax2* datasets, we abbreviated them in the results to *ECG1* and *ECG2*.

Throughout our analysis, we employed Wilcoxon tests for pairwise significance comparisons, chosen due to the non-normal distribution of the data and sample dependence. For all applied significance tests the significance threshold was set to $\alpha = 0.05$. To account for multiple comparisons, we adjusted the p-values using Holm-Bonferroni correction.

#### PM choice insights

This subsection provides insights influenced by PM choice, which we discuss in the following groupings:

- PM suitability across all investigated datasets, models and region sizes
- Differences in PM suitability between datasets, models and region sizes
- Impact of PM selection per class
- Model-specific observations
- Dataset-specific observations

**Fig. 4:**
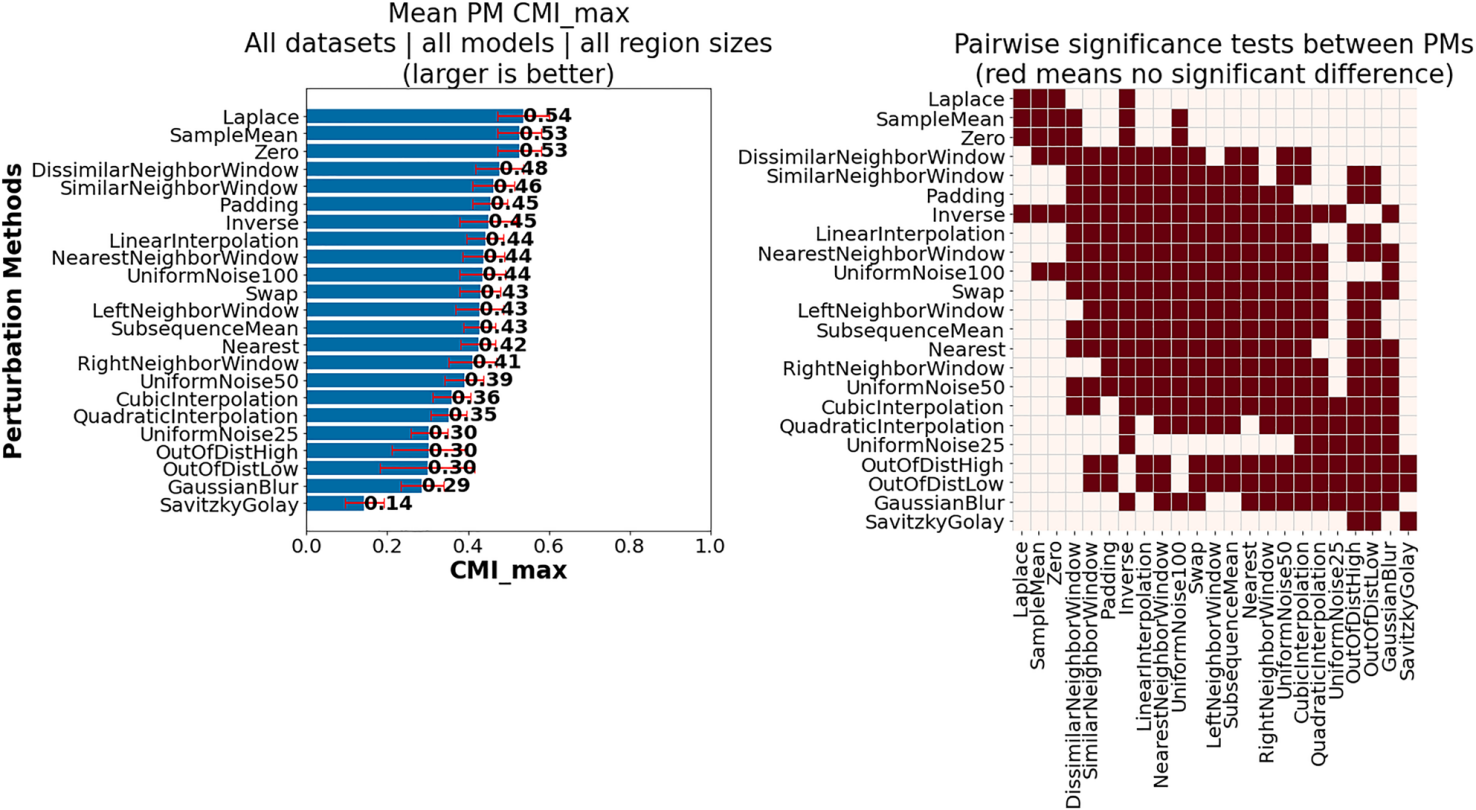
Comparison of highest achievable CMI for each PM across all datasets models and region sizes: (left) Mean $CMI_{max}$ for each PM; (right) Pairwise significance tests between PMs—Laplace, SampleMean and Zero best overall PMs.

*Investigation of PM suitability across all investigated datasets, models and region sizes: * Figure 4 presents the mean $CMI_{max}$ for each PM over all datasets, models and region sizes (left), including the outcome of pairwise significance tests (right). From this figure it is evident that one of the investigated PMs, Laplace, is among the best overall PMs, together with SampleMean and Zero, which have also previously been shown to generally work well. While we will show later that for many individual datasets and models other PMs are often better suited, this suggests that these three PMs can be utilized as default PMs, especially in cases where little information is available regarding the actual data generation process or task. Additionally, while there is no significant difference between the top three performing PMs and Inverse, we do not recommend it as a default choice, given that for individual cases that will be shown later it can perform very poorly. The same applies to UniformNoise100.

In contrast, smoothing approaches, i.e., GaussianBlur and SavitskyGolay, perform on average as badly or worse than even the two baseline PMs (OutOfDistHigh and OutOfDistLow). In retrospect, their bad performance is not unexpected, given that they preserve the actual trends in the data and remove the noise. However, these PMs may still prove useful for evaluating AM faithfulness on datasets where noise may be a distinguishing class specific property. Moreover, it can be seen that when relying on noise injection methods, it is generally better to inject more noise (UniformNoise100) than less (UniformNoise25).

*Finding PM1*: *Recommended default PMs—SampleMean, Zero and Laplace.*

*Finding PM2*: *Not Recommended PMs—SavitzkyGolay and GaussianBlur.*

*Differences in PM suitability between datasets, models and region sizes: * By comparing the individual PM rankings, we could observe that the most suitable PMs are not only dataset specific, but also dependent on the used model architecture and applied region size.

Table 8 shows the three most suitable PMs (out of 23) according to their $CMI_{max}$, for the same model architecture and region size, but different datasets. The mean and standard deviations are computed over the 5 seeds per model architecture. A stark difference in PM suitability between datasets can be observed. As expected, datasets that are similar in nature, e.g., ECG1 and ECG2, may have the exact same suitable PMs. However, even slight differences between datasets may cause strong impacts. While the FordA and FordB datasets deal with the same type of problem (symptom detection in working engines), the time series in the FordB dataset contain noise. This presumably lead the models to learn different aspects of the data, which lead to a different set of suitable PMs.

**Table 8:** Differences in most suitable three PMs—ranked by $CMI_{max}$—for the same model architecture (ResNet) and region size (2.5% input length), but different datasets, averaged over 5 model seeds. Optimal PM vary depending on dataset type. Very similar datasets may have same optimal PMs (ECG1 & ECG2), however small difference in datasets may results in different most suitable PMs (FordA & FordB).

Dataset	$CMI_{max}$ range across PMs		Perturbation method	$CMI_{max}$	
				Mean	SD
FordA	Max	0.4532	LinearInterpolation	0.4532	0.0187
	Min	0.0	Padding	0.4346	0.0165
	Mean	$0.33 \pm 0.13$	Swap	0.4277	0.0248
FordB	Max	0.5261	SubsequenceMean	0.5261	0.0356
	Min	0.0	SampleMean	0.5027	0.0576
	Mean	$0.37 \pm 0.15$	Zero	0.5027	0.0576
ECG1	Max	0.8133	LeftNeighborWindow	0.8133	0.0362
	Min	0.0029	DissimilarNeighborWindow	0.7748	0.0392
	Mean	$0.50 \pm 0.24$	NearestNeighborWindow	0.7723	0.0416
ECG2	Max	0.7184	LeftNeighborWindow	0.7184	0.0840
	Min	0.0031	DissimilarNeighborWindow	0.6950	0.0655
	Mean	$0.48 \pm 0.21$	NearestNeighborWindow	0.6864	0.0788
Wafer	Max	0.4598	Laplace	0.4598	0.1600
	Min	0.0	SampleMean	0.3977	0.1043
	Mean	$0.19 \pm 0.13$	Zero	0.3977	0.1043

Alternatively, Table 9 shows the three most suitable PMs for the same dataset and region size, but for different model architectures. Intuitively, one would assume, that for the same dataset, the same PMs would be fitting, which was not the case. It can be seen that in three cases SampleMean and Zero are highly ranked (Inception, MLP and ViT), while in two cases PMs which replace the values by using neighboring subsequences (LSTM and ResNet). Also, in this particular case, even though Inception and ResNet are both CNN-based models, and both achieve a very similar accuracy on the test set (~95%), they differ in PMs which are most suitable. We hypothesize that due to the architectural differences, specifically differently sized kernels, that different strategies emerged to detect the individual classes, resulting in different optimal PMs. This finding is of considerable importance, since it implies that the * data properties are not the exclusive driver that impact PM selection, instead the data properties the model* actually learned *to rely on*. This in turn means that perturbation approaches that rely on generative models trained directly on the data may still fail to provide adequate results for AM faithfulness evaluation.

**Table 9:** Differences in top three PMs for the same dataset (ECG1), same region size (2.5% input length) and different model architectures. Optimal PM dependent on model type.

Model	$CMI_{max}$ range across PMs		Perturbation method	$CMI_{max}$	
				Mean	SD
Inception	Max	0.7155	Zero	0.7155	0.0610
	Min	0.0095	SampleMean	0.7155	0.0610
	Mean	$0.44 \pm 0.24$	Laplace	0.7136	0.0601
LSTM	Max	0.772	NearestNeighborWindow	0.7720	0.0366
	Min	0.262	UniformNoise25	0.7510	0.0331
	Mean	$0.67 \pm 0.11$	DissimilarNeighborWindow	0.7450	0.0351
MLP	Max	0.8337	UniformNoise50	0.8337	0.0082
	Min	0.0086	SampleMean	0.7514	0.0076
	Mean	$0.58 \pm 0.22$	Zero	0.7514	0.0076
ResNet	Max	0.8133	LeftNeighborWindow	0.8133	0.0362
	Min	0.0029	DissimilarNeighborWindow	0.7748	0.0392
	Mean	$0.50 \pm 0.24$	NearestNeighborWindow	0.7723	0.0416
ViT	Max	0.7729	SampleMean	0.7729	0.0164
	Min	0.0122	Zero	0.7729	0.0164
	Mean	$0.58 \pm 0.20$	Laplace	0.7721	0.0165

Finally, Table 10 shows the three most suitable PMs for the same dataset and model, but different region size. While different PMs may be better suited depending on region size, increasing the region size yields overall smaller *CMI*. We speculate that by modifying too much of the input at once, many not relevant data aspects are perturbed as well, leading to an overall slower decline.

**Table 10:** Differences in top three PMs for the same dataset (ECG1), same model architecture (Inception) and different region sizes. Noticeable difference in $CMI_{max}$ and optimal PM depending on region size.

Region size (% of input length)	$CMI_{max}$ range across PMs		Perturbation method	$CMI_{max}$	
				Mean	SD
2.5%	Max	0.7155	Zero	0.7155	0.0610
	Min	0.0095	SampleMean	0.7155	0.0610
	Mean	$0.44 \pm 0.24$	Laplace	0.7136	0.0601
10%	Max	0.6209	CubicInterpolation	0.6209	0.0516
	Min	0.0076	Swap	0.5991	0.0836
	Mean	$0.34 \pm 0.19$	Zero	0.5029	0.0528

*Finding PM3*: *Best PMs specific to dataset, model and region size.*

*Finding PM4*: *Data properties are not the exclusive driver that impact PM selection, but the data properties the model*  *actually learned* *to rely on.*

**Fig. 5:**
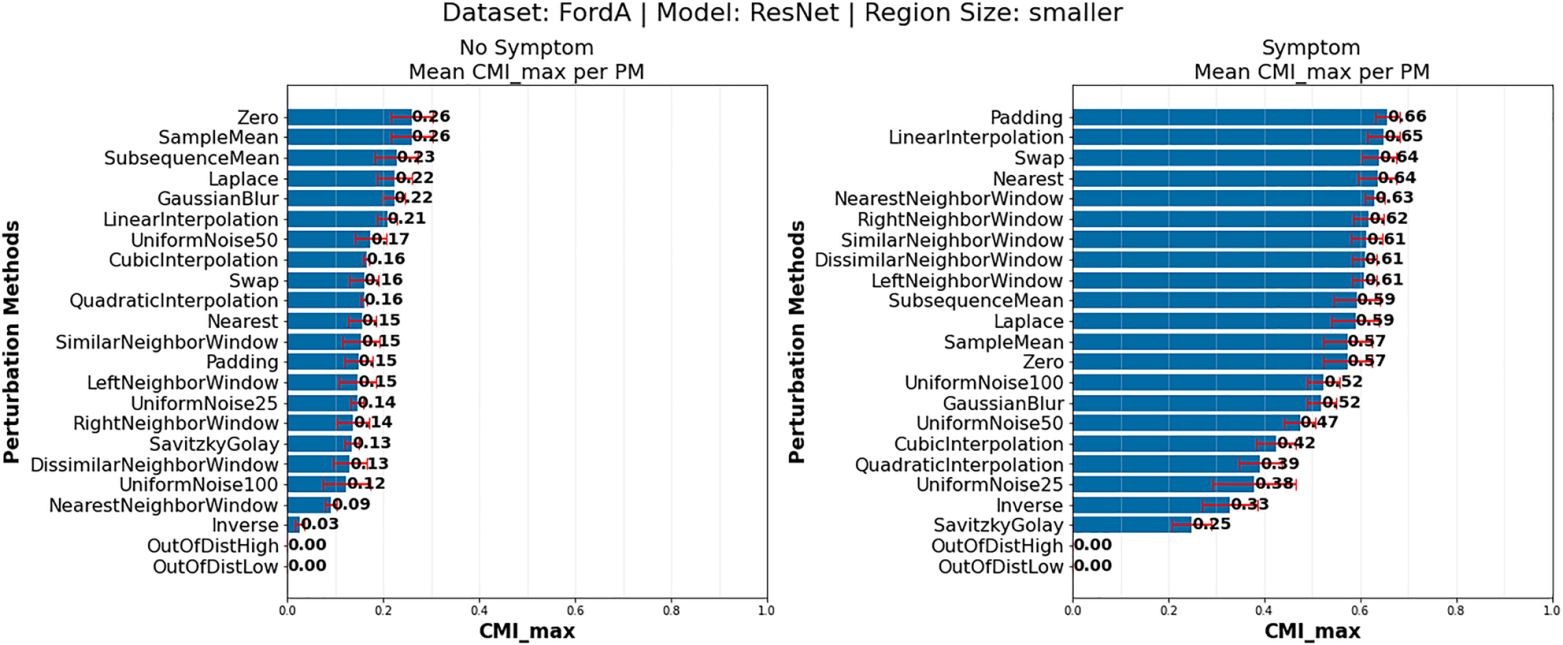
Comparison of highest achievable CMI over all AMs for each PM per class on the FordA dataset, smaller region size and ResNet architecture (5 models). Most suitable PMs for the *No Symptom* (left) and *Symptom* (right) class. PM suitability dependent on class. Also, a strong difference in $CMI_{max}$ between classes can be observed, due to number of relevant time points to detect the class.

*Investigation of PM effect for different classes: * Given that we have seen that different PM suitability can depend on dataset, model and region size, we additionally compared the differences between samples of different classes, for each dataset, model and region size combination. Unsurprisingly we observed here differences as well. Figure 5, shows the complete $CMI_{max}$ PM rankings of the FordA dataset, ResNet model architecture and smaller region size, for the “No Symptom” (left) and “Symptom” (right) class. Two notable observations can be made from the figure: (i) the difference in mean $CMI_{max}$ values, and (ii) the difference in PM ranking. The differences in the mean $CMI_{max}$ value come from the *number* of time points that are important for the model to make a prediction. The model relies on a smaller set of time points to detect a time series as *Symptom*, than as *No Symptom*. Therefore, the *MoRF* perturbation curve will decrease for *Symptom* on average rapidly after only a few perturbations, causing a larger *DDS* and therefore, increasing the *CMI*. The difference in the ranking of PMs on the other hand is likely due to the *nature* of the data aspects the model actually learned to rely to detect the class. Thus, different PMs are best suited to remove these prediction relevant aspects.

*Finding PM5*: *Different PMs better for different classes.*

**Fig. 6:**
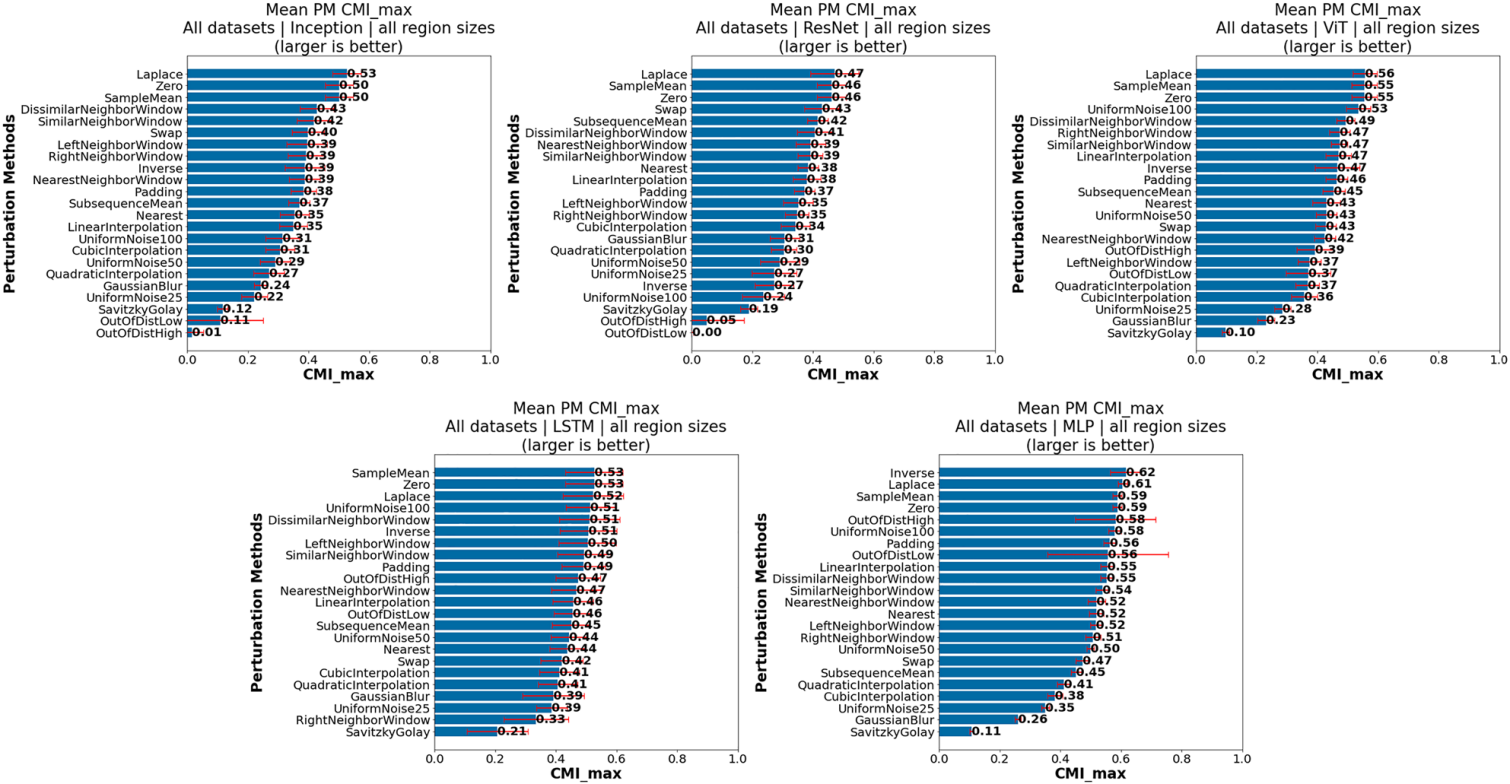
Ranking of most suitable PMs for every model architecture across all datasets and region sizes according to mean $CMI_{max}$—SampleMean, Zero and Laplace overall best for each model, whereas Inverse ranked high for LSTM and MLP. Variable suitability for other PMs depending on model architecture.

*Model specific observations: * To investigate model specific PM findings, we aggregated the $CMI_{max}$ values of each PM across all datasets and both region sizes (Fig. 6) per model architecture. Notably, across all models SampleMean, Zero and Laplace PMs performed best, which was expected given that these PMs, are generally the best when aggregating over all datasets and models. For the Inception, ResNet and ViT models, additionally the neighboring based PMs performed quite well. For LSTM all neighboring based PMs, except for RightNeighborWindow, performed also well. Given that an LSTM makes its predictions by parsing the time series from beginning to end (left to right), we assume that modifications of previous (left) values are taken more into account. Moreover, it can be observed that for the MLP and LSTM architectures strong PMs (e.g., Inverse, UniformNoise100, OutOfDistHigh and OutOfDistLow) are suitable. This effect is especially prominent with MLP, where the Inverse PM seems to be the most suitable, and where the baseline PMs (OutOfDistHigh and OutOfDistLow) PMs achieved very high mean $CMI_{max}$, although with a high variability. We hypothesize that the MLPs due to potential overfitting learned some spurious correlation, which the strong PM effect exploited. However, we did not confirm this hypotheses. It is notable that for ViT UniformNoise100 also worked quite well, while for the other vision models, Inception and ResNet, it did not.

*Finding PM6*: *Laplace, SampleMean and Zero PM recommended across all model architectures and region sizes. Variability for other PMs depending on model architecture.*

**Fig. 7:**
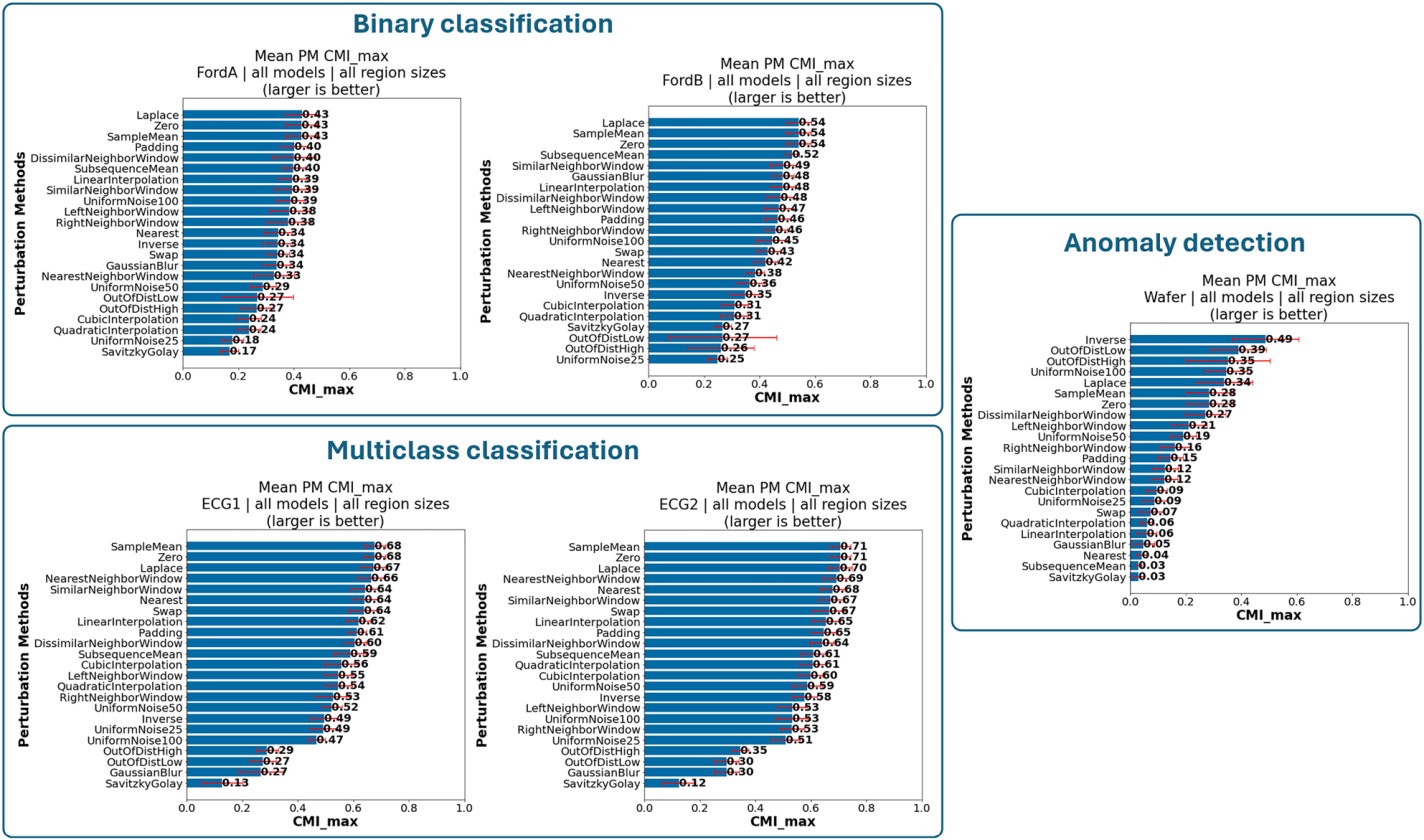
Ranking of most suitable PMs for each dataset across all models and region sizes according to mean $CMI_{max}$. Laplace, SampleMean and Zero PM recommended for *most* investigated dataset types. PMs that modify input strongly better suited for anomaly detection dataset.

*Dataset type specific PM findings* As previously mentioned, the datasets can be grouped into the following three groups: (i) balanced binary classification datasets (FordA, FordB); (ii) multiclass classification datasets (ECG1, ECG2); and (iii) imbalanced binary classification—anomaly detection (Wafer). Based on the three groups of datasets, we investigated the differences in suitable PMs based on the mean $CMI_{max}$ over all models and region sizes (Fig. 7). What immediately stands out is that for the Wafer dataset, which is an anomaly detection dataset, PMs which modify the input very strongly (i.e., Inverse, OutOfDistHigh, OutOfDistLow and UniformNoise100) are actually the most suitable for evaluating AMs, while the generally well suited PMs (*Laplace*, *SampleMean* and *Zero*) follow. Moreover, the distribution of mean $CMI_{max}$ across PMs is skewed, and there are high standard deviations, indicating strong differences between models. Regarding the balanced binary and multiclass classification datasets, we can observe that the distribution of $CMI_{max}$ over all PMs is not as skewed as for the Wafer dataset. It is notable that the generally recommended PMs are also the most suited for these datasets. What is also noteworthy is that while *GaussianBlur* works very badly for the multiclass classification datasets, it works far better for the balanced binary classification datasets, and especially the noise containing FordB dataset. This may be due to the nature of the datasets where the tasks for the FordA and FordB datasets consist of detecting symptoms in engines, which may be due to noise, which the PM effectively removes.

Moreover, there is a differences in the $CMI_{max}$ values between the different types of datasets, indicating again how much of the input is relevant for the prediction.

*Finding PM7*: *Laplace, SampleMean and Zero PM recommended for most investigated dataset types.*

*Finding PM8*: *PMs that modify input strongly better suited for anomaly detection dataset.*

*Summary of PM findings: * Through the performed experiments, we could confirm that the most suitable PMs are not only dependent on the used dataset, but also on the used models, applied region size, and can also be class specific for the same dataset. These findings strengthen the importance of PM selection when evaluating AMs. However, there are PMs (*Laplace*, *SampleMean* and *Zero*), which generally performed well across all investigated configurations. These PMs may be a good default choice when little information is available on the data generation process and used model, or may be used as a baseline for finding better and more suitable PMs.

#### AM insights

In this section, we will first present AMs that are generally recommended or should be avoided. Afterwards, we explore and discuss model-specific and dataset-specific AM findings.

**Fig. 8:**
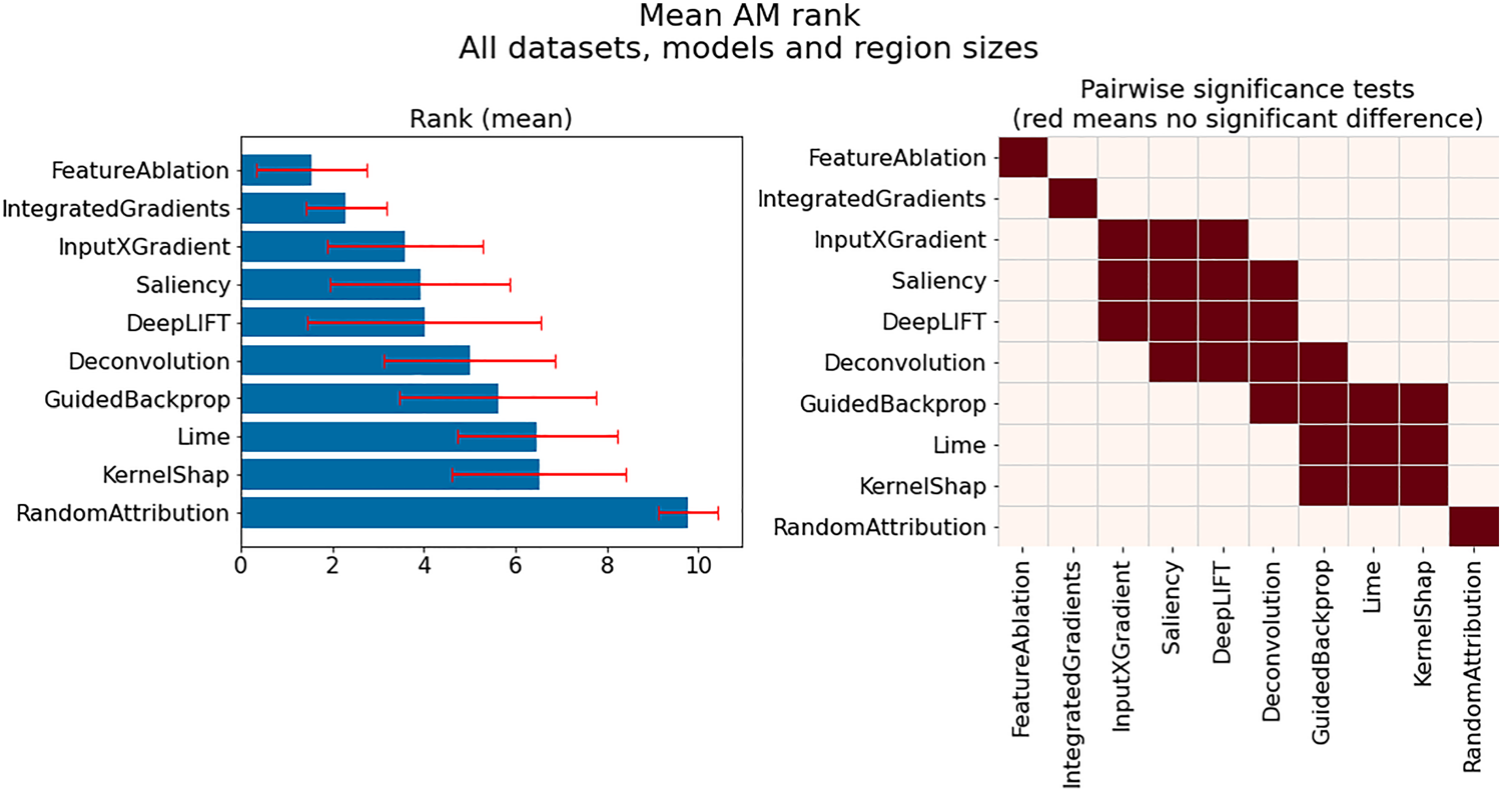
Mean AM ranks for all datasets, models and region sizes (left), and pairwise significance tests (right), exluding GradCAM and GuidedGradCAM—FeatureAblation provides the most faithful explanations, followed by IntegratedGradients.

**Fig. 9:**
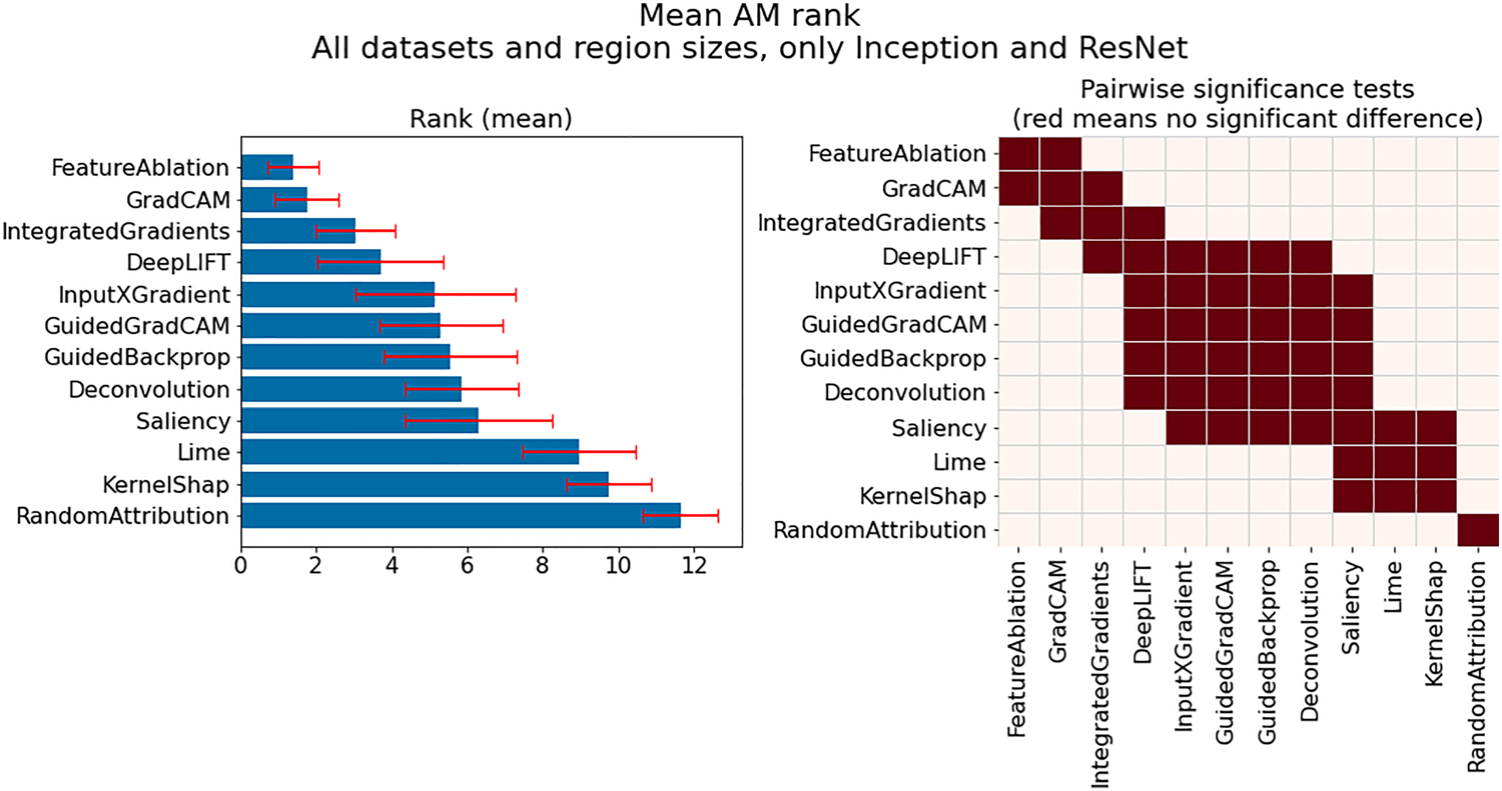
Mean AM ranks for all datasets and region sizes over ResNet and Inception model architectures, including GradCAM and GuidedGradCAM (left), and pairwise significance tests (right)—FeatureAblation and GradCAM provide the most faithful explanations for the investigated CNNs.

*Investigation of AM faithfulness across all investigated datasets, models and region sizes:* Figure 8 presents the ranking of AMs aggregated over all datasets, models, and region sizes. In this Figure GradCAM and GuidedGradCAM are excluded, since they are only available for CNNs. Figure 9 includes GradCAM and GuidedGradCAM, and aggregates the results over all datasets and region sizes, and only the CNN models, namely Inception and ResNet.

Excluding RandomAttribution—which was correctly ranked worst—the AMs can be roughly grouped into four faithfulness brackets:

- *Best:* FeatureAblation, GradCAM*
- *Good:* IntegratedGradients
- *Medium:* InputXGradient, Saliency, DeepLIFT, Deconvolution, GuidedBackprop, GuidedGradCAM*
- *Bad:* KernelSHAP, LIME

The AMs marked with an asterisk (*) are applicable only to CNNs. It is evident from the ranking that FeatureAblation can generally be recommended, especially since this is a model agnostic AM. It is also evident that GradCAM, where compatible, can be clearly recommended, especially given that it is model-specific method and generates explanations much faster than FeatureAblation. However, for use cases where explanations need to be generated very rapidly, and GradCAM cannot be used, IntegratedGradients presents a viable alternative.

The overall performance of KernelSHAP and LIME is subpar, thus we discourage the use of these AMs for explaining neural time series classification models that make predictions based on raw time series data.

*Finding AM1*: *Recommended default AMs—FeatureAblation and GradCAM.*

*Finding AM2*: *Not recommended AMs—KernelSHAP and LIME.*

*Importance of AM selection for model architectures: * Table 11 presents the mean rank of each AM per model architecture, aggregated over all datasets and region sizes. This table includes only AMs compatible with all model architectures. Notably, FeatureAblation emerges as the most faithful AM for all investigated model architectures, except for MLP, where DeepLIFT performs best. This suggests that Feature Ablation is robust and effective across different types of models. Additionally, IntegratedGradients (Inception, LSTM, ViT) and DeepLIFT (ResNet and MLP) generally provide faithful explanations. However, it is noteworthy that for ViT DeepLIFT performed exceptionally poorly, while Saliency performed better. Conversely, KernelSHAP, LIME, perform poorly across all model architectures, while GuidedBackprop performs badly for LSTM and MLP. Furthermore, RandomAttribution was correctly ranked as worst for all models.

Table 12 provides a separate ranking for ResNet and Inception that includes GradCAM and GuidedGradCAM. It can be seen that for both CNN-based models, GradCAM offers almost as faithful explanations as FeatureAblation. GuidedGradCAM on the other hand did not prove as faithful.

While FeatureAblation tends to provide the most faithful explanations and can be used with any model, it comes with the drawback that it is computationally very expensive, given that it has to probe the model repeatedly for each time series. Many other model specific AMs exhibit high variability in faithfulness across the investigated model architectures, stressing the importance of AM faithfulness evaluation.

**Table 11:** Mean AM ranks for all model architectures over all datasets and region sizes. GradCAM and GuidedGradCAM exluded, since they are not compatible with all models.

Atribution method	Inception	ResNet	LSTM	MLP	ViT
FeatureAblation	** 1.1 ± 0.32**	**1.3 ± 0.48**	**1.1 ± 0.32**	3.0 ± 2.0	**1.2 ± 0.63**
IntegratedGradients	1.7 ± 0.67	2.9 ± 0.32	1.9 ± 1.2	2.6 ± 0.7	2.4 ± 0.84
InputXGradient	3.4 ± 1.51	4.7 ± 1.34	4.5 ± 2.07	2.5 ± 1.35	2.8 ± 1.14
DeepLIFT	4.0 ± 0.82	1.8 ± 0.63	4.0 ± 1.83	**2.2 ± 1.14**	8.1 ± 1.45
GuidedBackprop	4.2 ± 1.87	4.4 ± 1.58	7.0 ± 1.49	6.0 ± 2.87	6.5 ± 1.35
Deconvolution	4.5 ± 1.51	4.5 ± 1.35	5.3 ± 1.34	6.1 ± 3.0	4.6 ± 1.43
Saliency	5.1 ± 1.79	4.6 ± 1.58	3.0 ± 2.62	4.1 ± 1.73	2.8 ± 1.03
LIME	6.7 ± 1.34	7.8 ± 0.79	6.2 ± 2.15	6.3 ± 2.36	5.4 ± 0.97
KernelSHAP	8.0 ± 0.94	7.8 ± 0.79	5.7 ± 2.06	5.5 ± 1.96	5.6 ± 1.71
RandomAttribution	9.9 ± 0.32	9.5 ± 1.08	10.0 ± 0.0	10.0 ± 0.0	9.5 ± 0.85

Best performing attribution methods are in [bold].

**Table 12:** Mean AM ranks for ResNet and Inception model architectures over all datasets and region sizes. Including GradCAM and GuidedGradCAM.

Attribution Method	Inception	ResNet
FeatureAblation	**1.2 ± 0.63**	**1.6 ± 0.7**
*GradCAM*	1.8 ± 1.03	1.7 ± 0.67
IntegratedGradients	2.3 ± 0.95	3.8 ± 0.42
InputXGradient	4.5 ± 2.22	5.8 ± 1.87
*GuidedGradCAM*	4.7 ± 1.77	5.9 ± 1.29
DeepLIFT	5.1 ± 0.57	2.3 ± 1.06
GuidedBackprop	5.6 ± 2.01	5.5 ± 1.58
Deconvolution	6.0 ± 1.56	5.7 ± 1.49
Saliency	6.7 ± 2.06	5.9 ± 1.85
LIME	8.5 ± 1.58	9.4 ± 1.35
KernelSHAP	9.8 ± 1.23	9.7 ± 1.06
RandomAttribution	11.9 ± 0.32	11.4 ± 1.35

Best performing attribution methods are in [bold].

*Finding AM3*: *Model architecture choice influences AM faithfulness.*

*Importance of AM selection regarding dataset types:* Table 13 presents the mean rank of each AM per dataset, aggregated over all model architectures and region sizes. This table includes only AMs compatible with all model architectures. Again, we found FeatureAblation to be the most faithful, delivering consistent results except for the anomaly detection dataset (Wafer). Although it’s noteworthy that the high standard deviation in this case might suggest the difference isn’t significant.

Integrated Gradients emerged as a close contender, demonstrating comparable faithfulness to FeatureAblation for the binary classification datasets (FordA and FordB) and multiclass classification datasets (ECG1 and ECG2). However, other AMs displayed considerable variations in performance. Saliency exhibited significant discrepancies. While it achieved a high rank for the multiclass classification datasets (ECG1 and ECG2), many other AMs produced more faithful results for the binary classification and anomaly detection datasets. Moreover, InputXGradient also provided mixed results. While it showed promise for binary classification and anomaly detection datasets, it demonstrated a poor performance for the ECG1 and ECG2 datasets. As expected from our previous results, KernelSHAP, LIME, showed a poor performance across all datasets.

If we solely consider the CNN models (Table 14, we notice that the faithfulness of GradCAM is comparable to FeatureAblation, which aligns with the results from our previous paper. GuidedGradCAM, again, did not prove as faithful.

It is evident from these results that most AMs do not perform uniformly across different datasets, suggesting that the *faithfulness of an AM is not only impacted by the model architecture, but also by the characteristics of the data as well*. Once again, these findings underscore the importance of selecting the appropriate AM depending on the used dataset and model.

**Table 13:** Mean AM ranks for all datasets over all model architectures and region sizes (best AMs in bold). GradCAM and GuidedGradCAM exluded, since they are not compatible with all models.

Attribution method	FordA	FordB	ECG1	ECG2	Wafer
FeatureAblation	**1.5 ± 1.08**	**1.9 ± 1.1**	**1.1 ± 0.32**	**1.0 ± 0.0**	2.2 ± 2.04
IntegratedGradients	2.2 ± 0.92	2.1 ± 1.1	2.7 ± 0.48	2.7 ± 0.67	**1.8 ± 0.92**
InputXGradient	2.7 ± 1.16	2.4 ± 1.07	5.2 ± 1.81	4.6 ± 1.17	3.0 ± 1.33
DeepLIFT	3.5 ± 2.55	3.7 ± 3.02	4.9 ± 2.51	5.0 ± 2.62	3.0 ± 1.76
Saliency	5.1 ± 0.74	6.2 ± 1.55	2.7 ± 1.49	2.4 ± 0.84	3.2 ± 1.75
Deconvolution	5.8 ± 1.14	5.3 ± 1.34	5.0 ± 1.94	5.2 ± 2.44	3.7 ± 1.89
KernelSHAP	6.5 ± 1.72	6.0 ± 1.25	6.6 ± 1.84	6.7 ± 1.95	6.8 ± 2.74
LIME	6.6 ± 1.35	6.6 ± 1.43	6.7 ± 1.57	6.7 ± 1.95	5.8 ± 2.49
GuidedBackprop	6.6 ± 1.26	6.5 ± 1.65	5.4 ± 2.32	5.6 ± 2.59	4.0 ± 2.0
RandomAttribution	9.6 ± 0.84	9.5 ± 0.97	10.0 ± 0.0	9.8 ± 0.63	10.0 ± 0.0

Best performing attribution methods are in [bold].

**Table 14:** Mean AM ranks for all dataset over ResNet and Inception model architectures and all region sizes (best AMs in bold). Includes GradCAM and GuidedGradCAM.

Attribution method	FordA	FordB	ECG1	ECG2	Wafer
FeatureAblation	**1.0 ± 0.0**	**2.0 ± 1.15**	**1.25 ± 0.5**	**1.25 ± 0.5**	**1.5 ± 0.58**
*GradCAM*	1.75 ± 0.96	2.25 ± 1.5	1.5 ± 0.58	1.5 ± 0.58	1.75 ± 0.5
IntegratedGradients	3.0 ± 1.15	2.75 ± 0.96	3.75 ± 0.5	3.5 ± 0.58	2.25 ± 1.5
DeepLIFT	3.5 ± 1.29	3.25 ± 2.06	4.25 ± 0.96	4.25 ± 1.5	3.25 ± 2.63
InputXGradient	4.25 ± 0.96	3.75 ± 1.5	8.0 ± 1.41	6.25 ± 1.26	3.5 ± 1.29
*GuidedGradCAM*	6.25 ± 1.5	6.5 ± 0.58	3.75 ± 0.5	5.0 ± 1.63	5.0 ± 2.16
Saliency	6.5 ± 1.0	9.0 ± 1.63	5.75 ± 1.89	4.25 ± 0.5	6.0 ± 0.82
GuidedBackprop	7.25 ± 1.71	6.5 ± 1.29	4.5 ± 1.73	4.25 ± 1.5	5.25 ± 0.96
Deconvolution	7.5 ± 0.58	6.25 ± 0.96	5.0 ± 1.41	4.25 ± 0.96	6.25 ± 1.26
LIME	8.5 ± 1.73	8.5 ± 1.91	10.0 ± 0.0	10.0 ± 0.0	7.75 ± 1.5
KernelSHAP	9.75 ± 0.5	8.0 ± 1.15	10.0 ± 0.0	10.25 ± 0.5	10.75 ± 0.5
RandomAttribution	12.0 ± 0.0	10.75 ± 1.89	12.0 ± 0.0	11.5 ± 1.0	12.0 ± 0.0

Best performing attribution methods are in [bold].

*Finding AM4*: *Dataset types influence AM faithfulness.*

*Summary of AM findings:* Generally, FeatureAblation stands out as the most faithful AM, while GradCAM can be recommended for compatible model architecture. Alternatively, IntegratedGradients can be recommended in cases where neither one of the previously mentioned AMs can be used, e.g., due to strict performance caps or model-AM incompatibility. However, for other AMs, especially DeepLIFT and Saliency, there exists a notable variability in their faithfulness rankings. We could observe that the AM rankings were influenced by both model architecture selection, as well as dataset characteristics. It is also evident that KernelSHAP and LIME should be generally avoided in the context of neural time series classification models utilizing raw time series data. Additionally, it’s noteworthy that the baseline AM consistently performs worst, validating the effectiveness of our AM faithfulness evaluation methodology.

#### Region size insights

This subsection investigates the impact of region size selection on:

- AM ranking
- PM ranking
- Model architecture specific PM ranking
- Dataset specific PM ranking

**Fig. 10:**
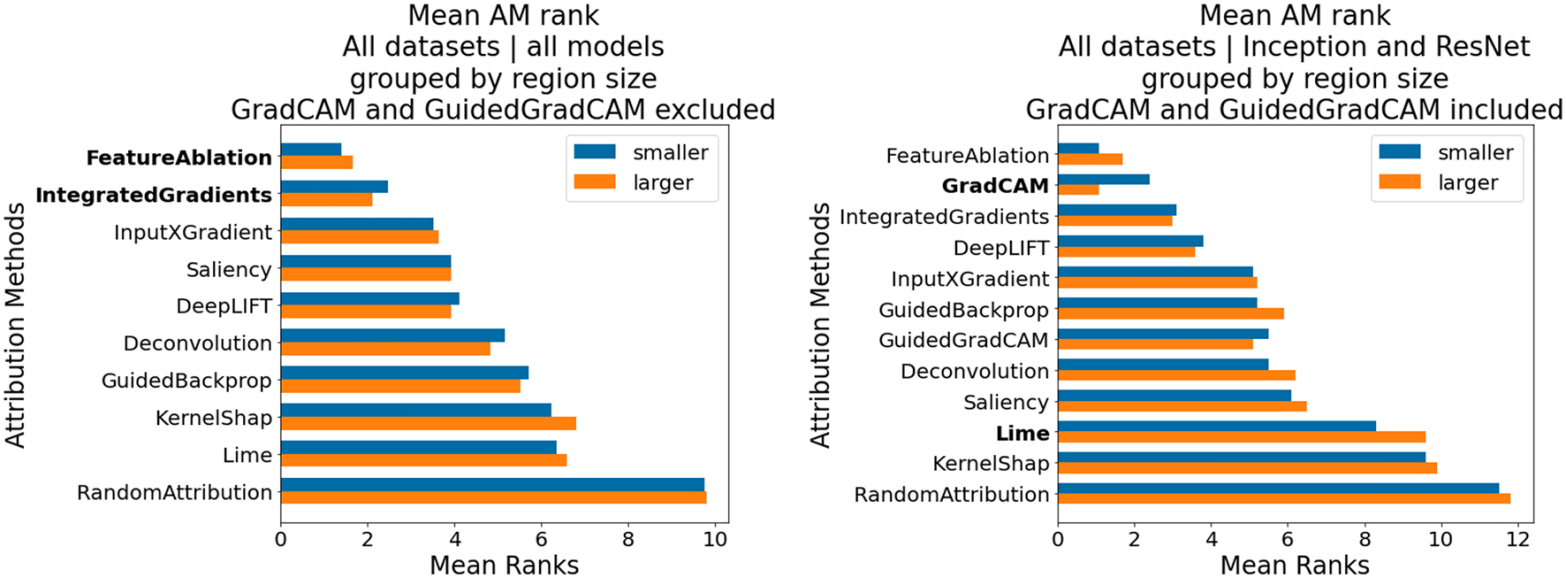
Comparison of mean AM ranks: (left) all datasets, models—GradCAM and GuidedGradCAM excluded; (right) all datasets, only Inception and ResNet models. For bolded AMs a there is a significant difference in rankings between smaller and larger region size. Almost no difference in AM ranking between smaller and larger region size. Only notable difference is GradCAM which is ranked best when a larger region size is used.

*Impact of region size on AM ranking:* Figure 10 depicts the mean rank of AMs compatible with all model architectures on the left, and the mean rank of all AMs for the CNN architectures, which includes GradCAM and GuidedGradCAM on the right. Generally, there is no difference in the AM rankings when smaller and larger region sizes are used to evaluate the faithfulness of AMs with our methodology. The only significant differences between the two region sizes is for FeatureAblation and IntegratedGradients, when aggregating over all datasets and models. However, even though there is a significant difference, both methods still achieve the highest and second highest rank when compared to other AMs.

However, for CNN model architectures only, there is crucial difference for GradCAM. First, there is a significant difference in the rankings, and second, this AM achieves the best rank, when a larger region size is applied.

Due to the adapted methodology, that incorporates PM suitability in the ranking, we expected little differences in the AM ranking. We hypothesize that the change in ranking for GradCAM is due to the fact, that GradCAM generally produces explanations that are “blurrier”, and therefore captures larger regions of importance better.

*Finding RS1*: *Minimal impact of region size on overall AM ranking with our methodology that accounts for PM suitability.*

*Finding RS2*: *GradCAM better when evaluated with larger region size.*

**Fig. 11:**
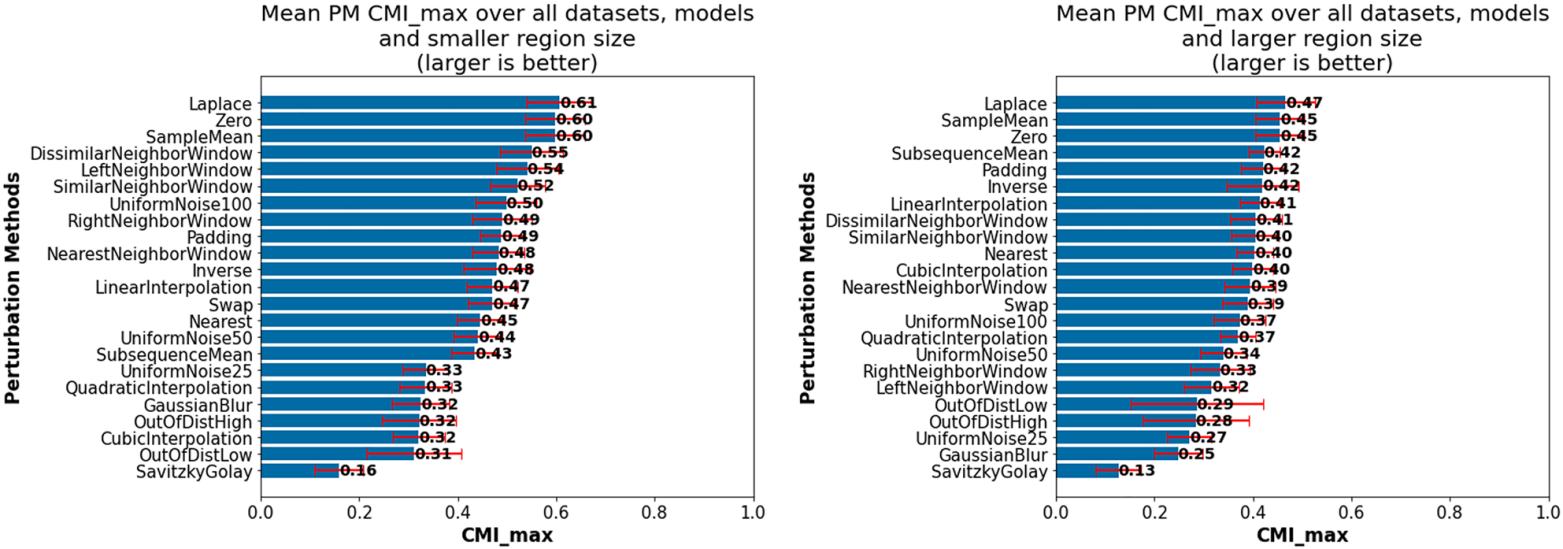
Mean $CMI_{max}$ ranks per PM across all datasets and models for smaller (left) and larger (right) region size. Larger region size yields overall smaller $CMI_{max}$ for most PMs.

*Impact of region size on overall PM ranking:* Figure 11 presents the region size comparison of mean $CMI_{max}$ for each PM, over all models and datasets. Given the overall PM recommendations from *Finding PM1*, it is not unexpected that Laplace, SampleMean and Zero are encountered as generally the best PMs also for each region size. However, it is noticeable that by using a larger region size, almost for every PM a smaller $CMI_{max}$ is achieved. The only exceptions are Cubic and QuadraticInterpolation, which work slightly better for larger region sizes.

It is also noticeable, that the NeighborWindow PMs are better for smaller region sizes, than for larger region sizes, where they are overtaken by other PMs.

Overall, we discourage the use of the SavitzkyGolay PM.

*Finding RS3*: *Laplace, SampleMean and Zero overall best for both region sizes.*

*Finding RS4*: *Large region size generally causes substantially smaller* $CMI_{max}$.

*Finding RS5*: *NeighborWindow PMs better for smaller region size.*

*Finding RS6*: *QuadraticInterpolation and CubicInterpolation improve with larger region size.*

*Impact of region size on PM ranking for model architectures:* Due to the large number of comparisons, the figures containing all the individual PM rankings per model architecture are provided in the Appendix. Generally, we noticed considerable variability in PM suitability across the region sizes for the investigated model architectures. We found that $CMI_{max}$ tends to decrease for larger region sizes across all individual model architectures. Particularly, the LSTM models exhibited the least noticeable change with an average decrease of 0.039 across all PMs, while for other model architectures the mean decrease ranged from 0.079-0.095. Despite these variations, SampleMean, Zero and Laplace PMs consistently demonstrated a good performance, either ranking highest or among the best across all individual model architectures and region sizes. Furthermore, we observed that NeighborWindow PMs generally perform better for smaller region sizes.

However, there are notable exceptions. Interpolation-based PMs, especially CubicInterpolation and QuadraticInterpolation, improve with larger region size. This is especially noticeable for Inception and ResNet, where CubicInterpolation improves drastically with larger region size. Thus, compared to other PMs, CubicInterpolation is well suited for larger region sizes, and comparable to Laplace, Zero and Samplemean for these architectures.

In contrast, for LSTM, strong PMs, specifically Inverse, OutOfDistHigh and Uniformnoise, perform best for larger region sizes, while SampleMean, Zero and Laplace for the smaller.

A similar trend is observed for MLP, where Inverse, Padding and LinearInterpolation perform much better for larger region sizes in comparison to other PMs. Additionally, SubsequenceMean shows considerable improvement for the larger region size.

*Finding RS7*: *LSTM* $CMI_{max}$ *impacted least by region size.*

*Finding RS8*: *PM suitability for ResNet, Inception and ViT robust to region size.*

*Finding RS9*: *PM suitability for LSTM and MLP strongly impacted by region size selection.*

*Impact of region size on PM ranking for individual datasets:* Once more, due to the large number of comparisons, we provide all individual PM rankings per dataset in the Appendix.

Most notably, substantial differences in $CMI_{max}$ are most prominent in the FordA and FordB datasets. The top-performing PM for the FordA dataset exhibits a mean $CMI_{max}$ approximately 0.32 higher for the smaller region size, with a similar difference of 0.22 for FordB. Conversely, for other datasets, the differences are far smaller and in the range from 0.04-0.06. Moreover, in addition to the reduction of $CMI_{max}$, considerable variability was observed among the top performing PMs per datasets depending on region size, showing the sensitivity of some datasets to choice of region size.

For instance, while SampleMean, Zero and Laplace PMs are best for FordB dataset independent of region sizes, for the similar FordA dataset there is a large difference between small and large region size. Specifically, NeighborWindow PMs are best for FordA when used in combination with the small region size, while for the large region size they should be avoided (drop in $CMI_{max}$ of  0.32).

Moreover, for the ECG1 and ECG2 datasets, the region size dependent PM behavior was very similar. For both datasets, there was a relatively small difference between the $CMI_{max}$ ranges across all PMs. However, it was notable, that the interpolation-based methods, specifically CubicInterpolation, performed quite poorly for the smaller region size, while it was best when the larger region size was used -CubicInterpolation $CMI_{max}$ improvement of 0.25 for ECG1 and 0.21 for ECG2.

Finally, for the Wafer dataset, we observed that the Inverse PM was best performing for both region sizes. It was also notable that the baseline PM OutOfDistLow achieved very good results for this dataset, likely due to the anomaly detection task. Most notably however was that the Neighbor PMs achieved better results for the larger region size for this dataset, while for the other datasets it was always the opposite.

*Finding RS10*: *PM suitability for most datasets strongly impacted by region size selection.*

*Summary of region size findings: * The findings demonstrate the robustness of our proposed methodology for ranking AMs based on their faithfulness across different region sizes. Notable differences emerged primarily in the reversed ranking of FeatureAblation and GradCAM for the CNN models. In terms of PM suitability, a consistent trend was observed toward higher $CMI_{max}$ values with smaller region sizes, with SampleMean, Zero, Laplace, as well as the NeighborWindow PMs exhibiting the best performance. Conversely, interpolation-based methods, notably CubicInterpolation and QuadraticInterpolation, demonstrated advantages with larger region sizes. Moreover, the findings also highlight variability in PM suitability across datasets and model architectures, emphasizing the need for careful consideration of region size. While our proposed AM ranking methodology remains robust against region size selection, employing smaller regions is recommended due to their tendency to yield higher $CMI_{max}$ values, thus ensuring more faithful results.

### Summary of findings & guidelines for AM faithfulness evaluation

The main findings from our experiments are summarized in Table 15. The results reveal the intricate relationship between PMs, dataset properties, model architectures and region size in the faithfulness evaluation of AMs.

**Table 15:** Summary of all findings depending on choice perturbation method and region size, as well as impact on attribution method faithfulness evaluation.

ID	Finding
PM1	Recommended default PMs—SampleMean, Zero and Laplace.
PM2	Not Recommended PMs—SavitzkyGolay and GaussianBlur.
PM3	Best PMs specific to dataset, model and region size.
PM4	Data properties are not the exclusive driver that impact PM selection, but the data properties the model *actually learned* to rely on.
PM5	Different PMs better for different classes.
PM6	Laplace, SampleMean and Zero PM recommended across all model architectures and region sizes. Variability for other PMs depending on model architecture.
PM7	Laplace, SampleMean and Zero PM recommended for most dataset types.
PM8	PMs that modify input strongly better suited for anomaly detection dataset.
AM1	Recommended default AMs—FeatureAblation and GradCAM.
AM2	Not recommended AMs—KernelSHAP and LIME.
AM3	Model architecture choice influences AM faithfulness.
AM4	Dataset types influence AM faithfulness.
RS1	Minimal impact of region size on overall AM ranking with introduced methodology.
RS2	GradCAM better when larger region size is used.
RS3	Laplace, SampleMean and Zero overall best for both region sizes.
RS4	Large region size generally causes substantially smaller $CMI_{max}$.
RS5	NeighborWindow PMs better for smaller region size.
RS6	QuadraticInterpolation and CubicInterpolation improve with larger region size.
RS7	LSTM $CMI_{max}$ impacted least by region size.
RS8	PM suitability for ResNet, Inception and ViT robust to region size.
RS9	PM suitability for LSTM and MLP strongly impacted by region size selection.

In the context of AM faithfulness evaluation for neural time series classification we found that there is no universally optimal PM. Rather, the results indicate that the best PM varies depending on the dataset, model architecture, region size used, and even *individual* classes within the dataset.

While certain PMs, specifically *Laplace*, *SampleMean*, and *Zero*, perform well across a wide range of experimental configurations, it is crucial to note that in many individual cases they are outperformed by other PMs. Although SampleMean and Zero have been used in AM faithfulness evaluations in previous literature without adequate justification, our results provide concrete evidence that these two PMs, along with our newly introduced Laplace PM, can serve as reliable default choices, particularly when limited information is available about the data generation process and the employed models. Furthermore, these can serve as comparisons for assessing the suitability of other PMs, potentially leading to the discovery of more appropriate alternatives. Therefore, for future AM faithfulness evaluations with our proposed methodology on time series data, we recommend the inclusion of the Laplace PM and SampleMean or Zero PM in addition to a small diverse set of other PMs, given that relying on single arbitrarily chosen PMs may lead to wrong conclusions.

In terms of AM findings, FeatureAblation emerged as the most faithful AM, offering robust performance across the investigated datasets and model architectures. GradCAM can be recommended for CNN model architectures, while IntegratedGradients presents itself as a viable option in cases where the other AMs may not be applicable due to performance constraints or model-AM incompatibility.

Our analysis also revealed considerable variability in the rankings of AMs in individual cases, influenced by factors such as model architecture and dataset characteristics. Notably, while some AMs may work reliably for most model architectures, such as DeepLIFT, they may provide unreliable results for a single model architecture like ViT. Conversely KernelSHAP and LIME exhibit poor performance across all investigated model architectures. Therefore, when choosing an AM for analyzing the behavior of a model, it is essential to consider as many AMs as possible to ensure the most faithful explanations in practice. Accordingly, we advise XAI researchers and practitioners from deriving general superiority of an AM method based on individual dataset-model combination, even though the AM may perform exceptionally well in a specific context.

Our experiments provided insights into the impact of region size on PM suitability and AM faithfulness evaluations. We observed a consistent trend towards higher effectiveness of certain PMs, such as SampleMean, Zero, and Laplace, with smaller region sizes. Conversely, interpolation-based methods like CubicInterpolation and QuadraticInterpolation show advantages with larger region sizes. Additionally, our findings indicate that employing smaller region sizes led to higher faithfulness scores. Consequently, we advocate for the use of smaller region sizes in the evaluation of AMs, as they have a higher potential to provide more faithful results.

Finally, we demonstrated the advantages of our proposed metrics and AM faithfulness evaluation methodology. Firstly, our metrics and methodology consistently and reliably reflected the poor performance of the baseline AM. By implementing a weighted ranking methods of AMs depending on PM and region size performance, we could significantly mitigate the negative effects of poorly chosen PM and region size combinations while amplifying the effects of effective ones.

In summary, the study presented a comprehensive analysis of the interplay between PMs, AMs, and various experimental parameters. By highlighting the importance of PM selection, elucidating the performance of different AMs across diverse scenarios, and exploring the impact of region size on PM suitability, it provides valuable insights for researchers and practitioners engaged in the faithfulness evaluation of AMs and explainability of neural time series classifiers.

## Practical example: AM faithfulness evaluation for a multivariate time series classifier

To illustrate the practical utility of our proposed AM faithfulness evaluation methodology, we present a user scenario focusing on understanding a neural time series classification model using XAI. This user scenario demonstrates the complete pipeline from identifying the most faithful AM to interpreting individual predictions of the targeted classifier for a multivariate time series dataset.

### Setup

Consider a data scientist tasked with training a classifier for recognizing epileptic seizures. The classifier should be trained on data collected by a triaxial accelerometer worn on the right hand of a person. In this example, the publicly available *Epilepsy* dataset from the UEA archive^65^ is used. The dataset consists of time series with three channels, namely the acceleration of the x, y and z-axis. Besides the epileptic seizure, the model should distinguish if a person is currently walking, running or sawing.

After training a well-performing classifier which can identify the four classes, the data scientist wants to understand if the model learned to rely on sensible patterns in the data. For this task, AMs are perfectly suited. However, before anything can be explained, an AM has to be selected, which in turn leads to the question: *“Which one is the best for this dataset and model?”*

To select the most faithful AM, the data scientist wants to apply our methodology. However, before the AM faithfulness evaluation can be performed, several decisions have to be made regarding: (i) AMs, (ii) PMs, (iii) Region Size, and (iv) Samples.

*AMs:* Initially, a set of relevant AMs has to be compiled, from which the most faithful one should be determined. This set may be influenced by the AM compatibility with the provided model, or if there is a white-box access to the model (i.e., access to the model’s code and weights). Given that the data scientist has access to the model, and has no performance requirements of the AMs, the same set of AMs as in the “Experimental setup” Section, excluding GradCAM and GuidedGradCAM due to incompatability with the provided model, as well as RandomAttribution.

*PMs:* Next, a set of PMs for the faithfulness evaluation has to be selected. As previously mentioned, it is best to select a small set of diverse PMs. Based on the results from the “PM choice insights” section, the following list of diverse PMs that work well for different datasets has been selected: Zero, Laplace, UniformNoise100, Padding and GaussianBlur.

*Region size:* While a higher region size speeds up the evaluation, it provides less reliable results. This parameter will be set to 2.5% of input length.

*Samples:* Finally, the samples which should be used in the evaluation have to be selected. Since the data scientist wants to understand if the model relies on sensible patterns for never seen data, he will use the test set of the dataset he is working with.

Now that the AMs, PMs, Region Size and Samples, have been selected, the AM faithfulness evaluation can be performed as described in the “Methodology” Section. However, given the use of multivariate time series data, slight modifications are necessary in the actual method for perturbing regions. For univariate time series, the time point relevances of a time series were divided in subsequences of length defined by region size, for which the region mean was computed. The same is done for multivariate time series, but for each channel separately. Following, the regions are perturbed from the most to least relevant one, across all channels. Similarly, if one would have an image dataset, the region size would represent the relative width and height of a patch that should be perturbed in the image.

Besides this small modification, the rest of the AM faithfulness evaluation is performed in the same manner.

### Results

**Fig. 12:**
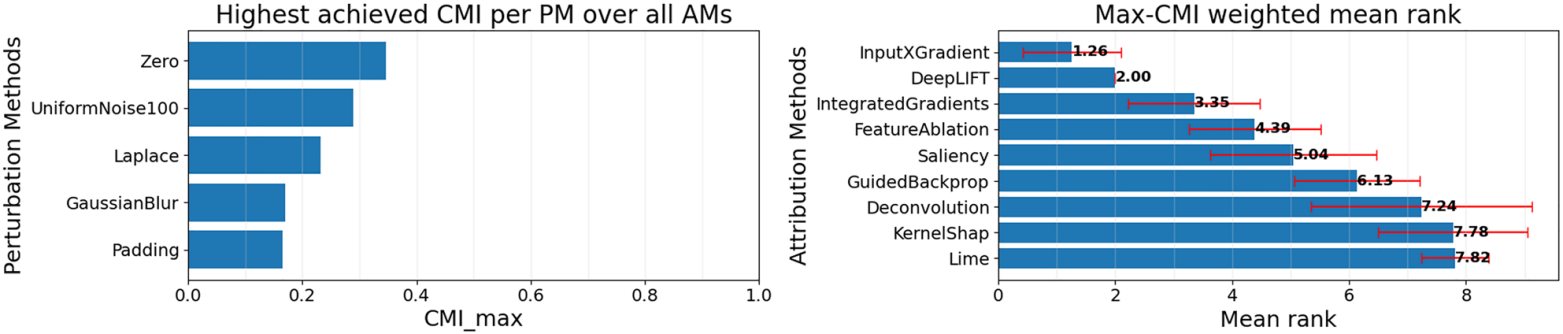
Outcome of faithfulness evaluation on multivariate time series dataset: (left) Per PM $CMI_{max}$over all AMs. (right) Mean AM rank.

Figure 12 (left) depicts the suitability of the PMs for evaluating the AMs on the epilepsy detection dataset and model. It is notable, that high CMIs are achievable with both the Zero and UniformNoise100 PMs. This means that these PMs remove the prediction relevant aspects in the input the best, without causing a significant shift in the distribution of the data. From Fig. 12 (right) it is evident that InputXGradient is the most faithful AM, while DeepLIFT was consistently ranked second. Both of these AMs were also ranked among the most faithful for the investigated univariate time series datasets, especially for the FordA and FordB datasets, which also consist of periodic time series patterns.

**Fig. 13:**
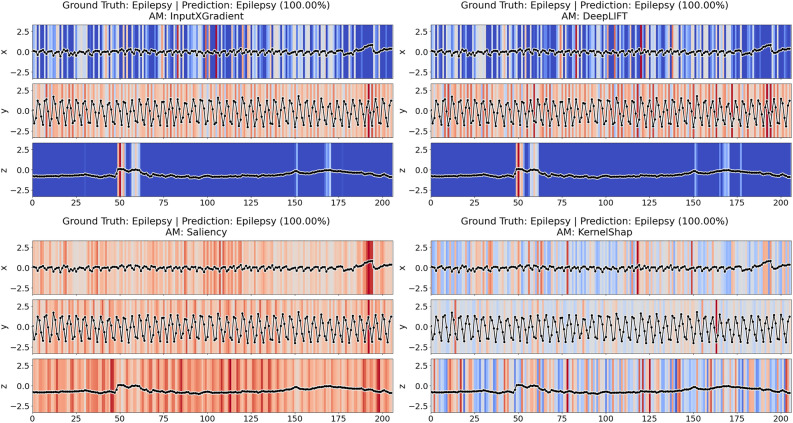
Comparison of explanations from differently ranked AMs for the same time series: InputXGradient (top-left) and DeepLIFT (top-right) are ranked as best and provide very similar explanations. Saliency (bottom-left) ranked in the middle, provides noisier explanations, while KernelSHAP (bottom-right) generates very noisy explanations.

Figure 13 shows examples of explanations provided by differently ranked AMs for the same time series sample. It is evident that the closely ranked AMs InputXGradient and DeepLIFT provide very similar explanations, while the explanation of Saliency (mean-rank=5.04) is noisy and KernelSHAP (mean-rank=6.92) much noisier. Accordingly, the data scientist would pick InputXGradient to understand the trained model.

**Fig. 14:**
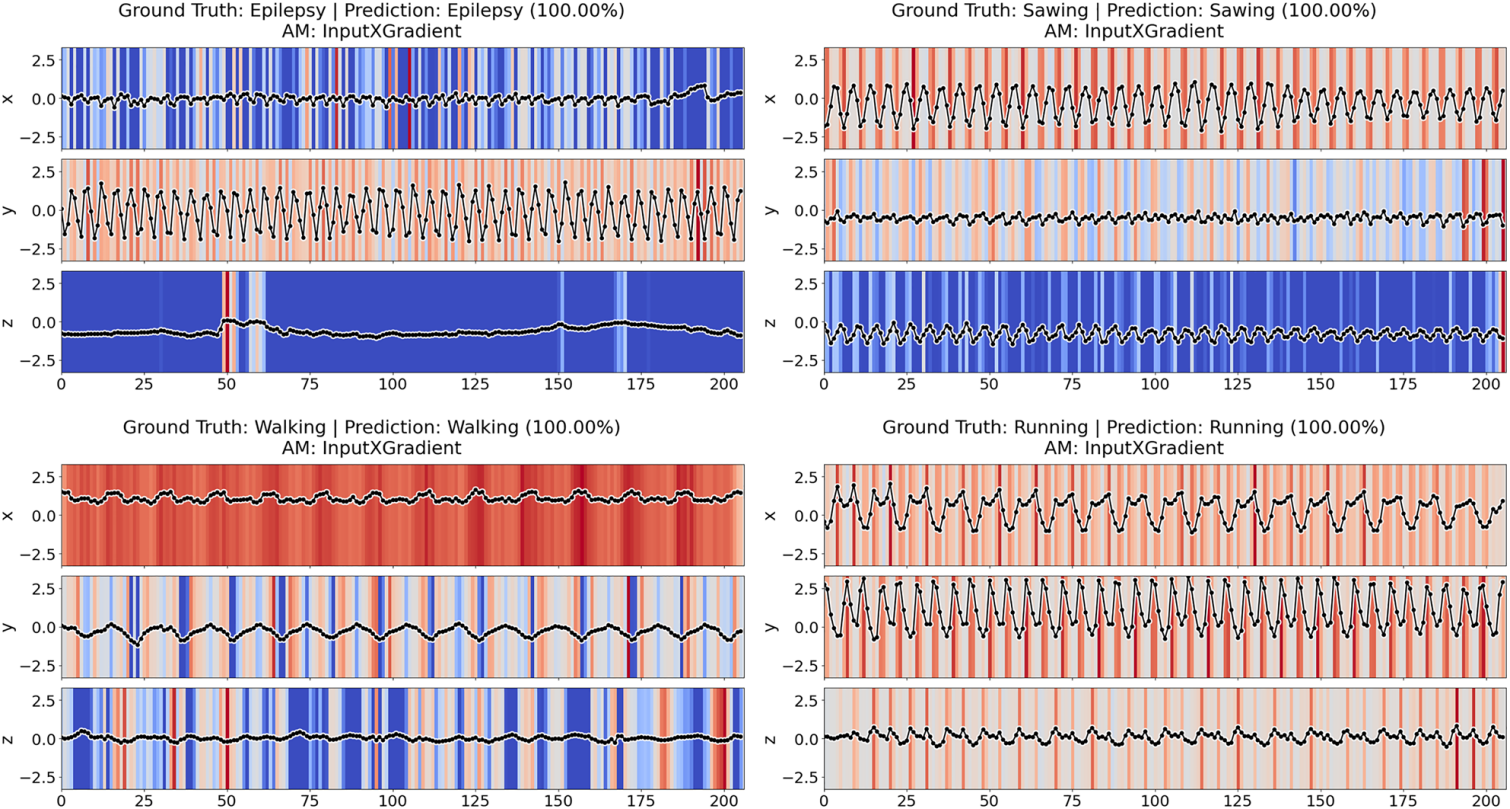
Comparison of explanations for different classes: epilepsy—strong fluctuations in *y* channel (top-left), sawing—strong fluctuations in *x* channel (top-right), walking—weak fluctuations in *x* channel (bottom-left), and running—strong fluctuations in *x* and *y* channel and weak fluctuations in *z* channel(bottom-right).

With InputXGradient the predictions of each class can be now faithfully explained to understand on what the model relies on. Figure 14 depicts a representative sample of each class, where the model made a confident prediction, including an explanation in the form of a heatmap. It is apparent that *epilepsy* (top-left) was detected mainly due to prolonged rapid strong changes in acceleration of the y-axis, while for *sawing* (top-right), rapid strong changes in acceleration of the x-axis where most important. On the other hand, for *walking* (bottom-left) constant small accelerations changes are prediction relevant. Finally, to recognize *running*, the model seems to consider activities from all three axes of the accelerometer. All of the explanations seem plausible to the data scientist. In a similar manner, the data scientist can further analyze other representative samples of the data, to detect potential errors in edge cases.

In conclusion, this practical example showcases how our methodology enables data scientists to identify the most faithful AM, facilitating a deeper understanding of what models learned. Additionally, the applicability of our methodology on multivariate time series data has been shown, emphasizing its easy transferability to other data types.

## Limitations and future work

In our work, we introduced a novel metric and evaluation methodology for assessing AM faithfulness in time series. However, certain limitations should be acknowledged. Addressing these limitations offers valuable context for researchers and practitioners interested in applying our methods and highlights opportunities for future research to refine and expand upon it.

- *Computational complexity:* A noteworthy drawback of the proposed approach is its computational complexity. Evaluating the faithfulness of a single AM with a PM requires systematic perturbation of each sample in the dataset. For instance, with a region size of 2.5% of the input length, generating the necessary perturbation curves for a single sample involves 40 perturbations (20 in MoRF order and 20 in LeRF order), each followed by model predictions. As the model size, dataset scale, and number of evaluated AMs increase, this process becomes increasingly time-consuming. Moreover, computation time grows linearly with each additional PM, and using multiple PMs is recommended for a robust evaluation. To address this issue, developing methods for reducing computation time, such as by identifying a core set—a small but representative data subset—would be highly beneficial. This is especially important, since we have shown that for different dataset and model combinations different AMs may be more faithful.
- *PM selection:* In our experiments, we thoroughly evaluated a large set of PMs. However, using all available PMs is impractical for real-world applications due to the substantial increase in computation time with each additional PM. In the “Practical example: AM faithfulness evaluation for a multivariate time series classifier” section, we addressed this challenge by empirically selecting a small, diverse subset of PMs based on the prior experimental findings. Future work could focus on developing systematic approaches to identify the most suitable PMs in advance, guided by the specific characteristics of the dataset and model. This would significantly reduce the computational burden of AM faithfulness evaluations.
- *Underexplored effects of model performance on AM evaluation:* The sanity check on the untrained model demonstrated that the introduced *CMI* metric responds appropriately under these conditions. However, since most AM faithfulness evaluations are conducted with well-trained models, it would be valuable to investigate how gradual changes in model performance influence the suitability of PMs and the resulting AM faithfulness assessments.
- *Methodology limited to classifiers: * The proposed methodology is currently limited to classifiers because it depends on changes in prediction confidence for the originally predicted class, which is in the range of [0,1]. This reliance makes it incompatible with regression models, which do not produce class probabilities. Adapting the approach to regression models would be a highly valuable extension for future work, as it would significantly broaden the methodology’s applicability. This could enable the evaluation of AMs in tasks involving continuous predictions, such as financial forecasting or industrial predictive maintenance.

As additional future work, a comparative study of PMs against generative imputation techniques, such as Generative Adversarial Networks or Variational Autoencoders, could shed light on which approach is superior, given that we have seen that data properties are not the sole driver of PM suitability.

Since the introduced approach is entirely model- and data-agnostic, it should generalize well to other data types. Applying this methodology to evaluate AMs and PMs across different models for image, text, and tabular data would provide valuable insights, particularly in identifying potentially misleading results that may have been overlooked in existing studies.

In our work, we adapted and extended the feature perturbation methodology of Samek et al.^3^, where we systematically perturbed time series subsequences with different PMs based on their relevance. To gain a deeper understanding of how alternative feature perturbation methodologies, such as those proposed by Schlegel et al.^48^ or Turbé et al.^52^, impact AM faithfulness evaluation—and the role of PM selection within these approaches—a comparative study could be conducted.

## Conclusion

Our study presented the critical role of PM selection in perturbation-based AM faithfulness evaluation, in particular concerning the interplay between the selected model architecture and dataset characteristics.

We have, once again, emphasized the importance of incorporating low relevance features in the AM faithfulness metrics. Subsequently, we introduced a novel metric, the *Consistency-Magnitude-Index*, which reliably estimates PM suitability for a given dataset and model, and how in combination with the proposed methodology, allows for a robust AM faithfulness evaluation.

Our extensive experiments involved applying region perturbation 27 600 times across all samples of the investigated 5 datasets, encompassing 5 deep learning model architectures with 5 models each. We evaluated 12 AMs with 23 PMs and 2 region sizes, over 2 perturbation orders. To the best of our knowledge, this represents the most comprehensive investigation to date on the impact of diverse PMs and region sizes on AM faithfulness evaluation for time series.

Our findings indicate the necessity for careful PM selection, as suitability varied significantly across model architectures, datasets, and even within different classes of the same dataset. Notably, we observed that data properties are not the exclusive driver that impact PM selection, but the data properties the model *actually* learned to rely on. This finding challenges the idea of using generative approaches as PMs.

SampleMean, Zero and the newly introduced Laplace PM serve as reliable default choices, across a range of models and datasets. Despite the fact that we recommend these PMs, in individual cases other PMs can outperform them. Therefore, to increase the robustness of AM faithfulness evaluation we proposed a methodology that utilizes a set of diverse PMs. Notably, PMs perturbing input based on neighboring regions provided promising results.

We raise awareness concerning faithfulness evaluations that rely on the also commonly used PM UniformNoise, as its effectiveness varies strongly across datasets and model architectures, potentially leading to misleading results.

Regarding AM selection, FeatureAblation generally provided the most faithful explanations across the investigated datasets and model architectures, while GradCAM is the suggested alternative for compatible model architectures, i.e. CNNs. IntegratedGradients offers an alternative to FeatureAblation in cases where execution time is of priority. Contrarily, GuidedBackprop, KernelSHAP and LIME should be avoided for neural time series classification models working with raw time series data.

Our proposed methodology and *CMI* metric offer broad applicability, demonstrated through a practical example on a multivariate time series dataset and can be extended to other data types.

For AM developers, the proposed methodology facilitates validation of newly developed AMs, while practitioners can confidently identify the most faithful AM for their specific model and dataset, aiding them in model debugging and validation.

## Supplementary Information

Supplementary Information.

## Data Availability

The data used in the experiments of this work and their respective data repository have been cited in the relevant sections. The code used in the experiments is publicly available under the following URL: https://github.com/perturbationeffect/cmi-am-validation-for-dl-ts-classifiers. Inquires regarding the data and the code can be sent to the author, Ilija Šimić.
