# Supplementary material for: A comprehensive analysis of perturbation methods in explainable AI feature attribution validation for neural time series classifiers
Source: Sci Rep. 2025 Jul 22;15:26607. doi: 10.1038/s41598-025-09538-2 (PMC12284047; doi:10.1038/s41598-025-09538-2)
Supplement: Supplementary file 1 — Supplementary Information. [file 41598_2025_9538_MOESM1_ESM.pdf]

Appendix

Model Architecture Descriptions

LSTM

The LSTM model consists of an LSTM layer and softmax classifier. The LSTM layer has 256 neurons. The softmax classifier is a fully connected layer where the number of neurons is equal to the number of classes in the dataset.

MLP

The MLP consists in total of four fully connected layers. The input layer, two hidden layers and a softmax classifier as the output layer. The first and second layer consist of 256 neurons, the third of 128 neurons, and the output layer has as many neurons as the datasets contain classes. Each of the 3 first layers is followed by a ReLU activation and Dropout layer set to 0.5.

Inception

The Inception model (adapted from Fawaz et al.[*]) consists of two residual blocks followed by a GAP layer and a softmax classifier for the output. The individual residual blocks consist of 3 inception blocks, where each inception block is followed by a ReLU activation. Before the last ReLU activation, the input of the residual block is added to the output of the last inception block. The inception blocks consist of a bottleneck layer, followed by 3 parallel convolutions of different kernel sizes and a parallel max pooling layer. The bottleneck layer drastically reduces the dimensionality of the input by performing a convolution with kernel size 1, stride 1 and 32 filters. The three parallel convolutions use kernel sizes of 41, 21 and 11. The output of the parallel convolution and max pooling layers are concatenated and a batch normalization is performed. The number of filters for all convolutional layers, except the bottleneck is set to 128.

ResNet

The ResNet model (adapted from Fawaz et al.[†]) consists of three residual blocks followed by a GAP layer and a softmax classifier as the output. Every residual block is composed of three convolutions. Each convolution is followed by a batch normalization and ReLU activation. Before the last ReLU activation, the residual block's input is added to the output of the batch normalization. The number of filters for all convolutions is set to 64. The filter length of the first convolution is set to 7, for the second to 5 and for the third to 3.

Vision Transformer (ViT)

For a detailed explanation of the Vision Transformer (ViT) architecture, please refer to the work of Dosovitzky et al.[‡]. Similarly to how CNNs are adapted for time series classification, the Vision Transformer (ViT) was adopted in such way, that the time series were treated as 1D images. As such, the height of the input "image" and the patches "image" was set to 1. The used ViT consisted of 6 transformer blocks, with 16 heads. The embedding layer and the classification head consist of 128 neurons. The dropout layers were set to 0.2. On top of the classification head, a softmax classifier is placed which has as many neurons as the datasets contain classes.

[*] Ismail Fawaz, H., Lucas, B., Forestier, G., Pelletier, C., Schmidt, D.F., Weber, J., Webb, G.I., Idoumghar, L., Muller, P.A. and Petitjean, F., 2020. Inceptiontime: Finding alexnet for time series classification. Data Mining and Knowledge Discovery, 34(6), pp.1936-1962.

[†] Ismail Fawaz, H., Forestier, G., Weber, J., Idoumghar, L. and Muller, P.A., 2019. Deep learning for time series classification: a review. Data mining and knowledge discovery, 33(4), pp.917-963.

[‡] Dosovitskiy, A., 2020. An image is worth 16x16 words: Transformers for image recognition at scale. arXiv preprint arXiv:2010.11929.

Impact of Region Size on Model Architecture PM Suitability

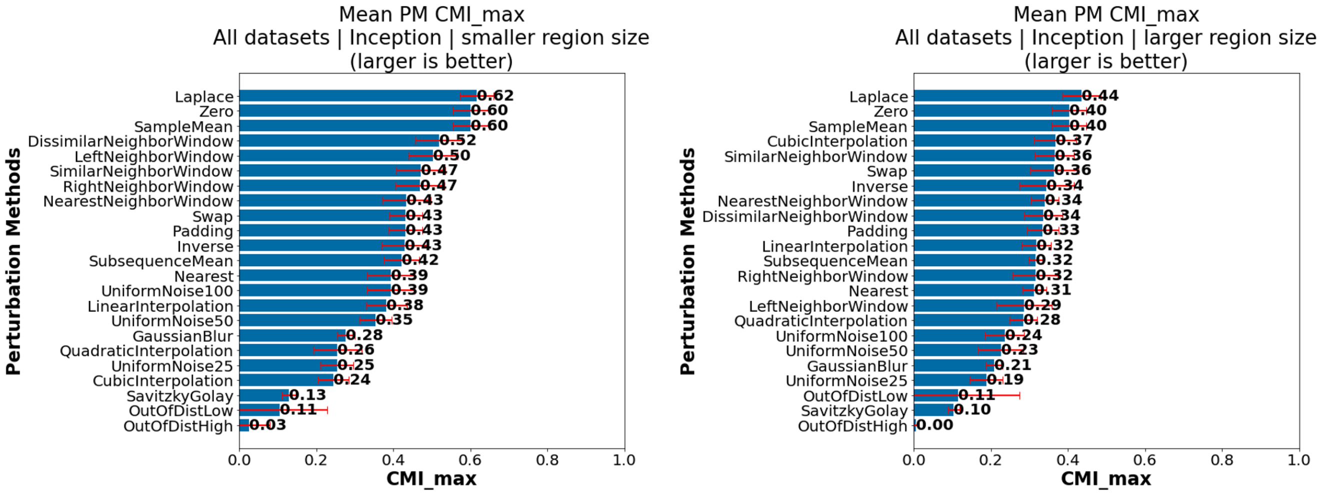

Supplementary Figure 1. Mean CMI_{max} per PM for Inception model, across all datasets - smaller region size (left), larger region size (right).

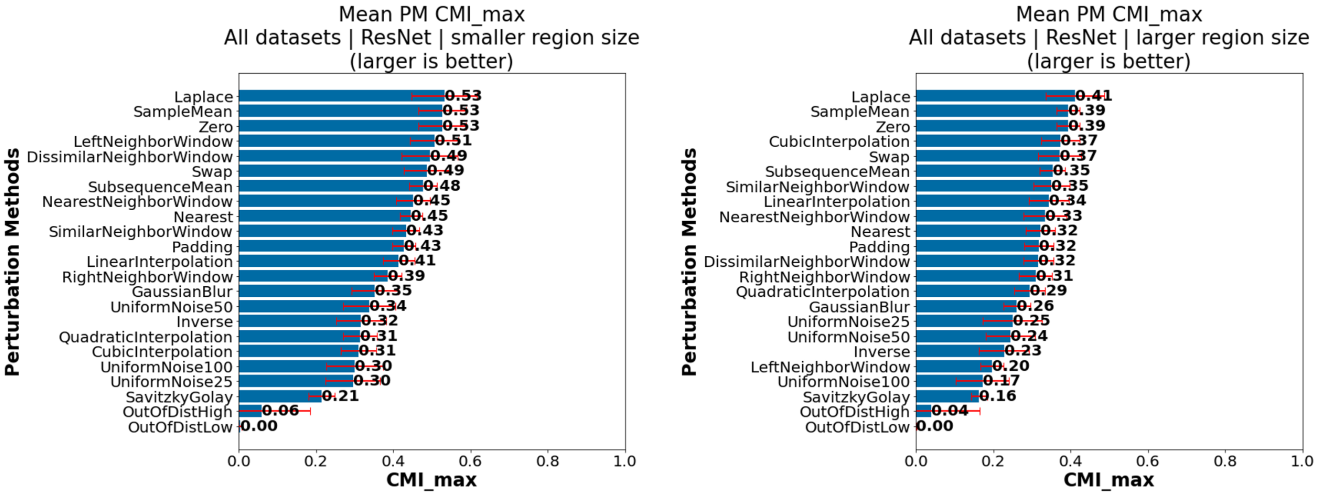

Supplementary Figure 2. Mean CMI_{max} per PM for ResNet model, across all datasets - smaller region size (left), larger region size (right).

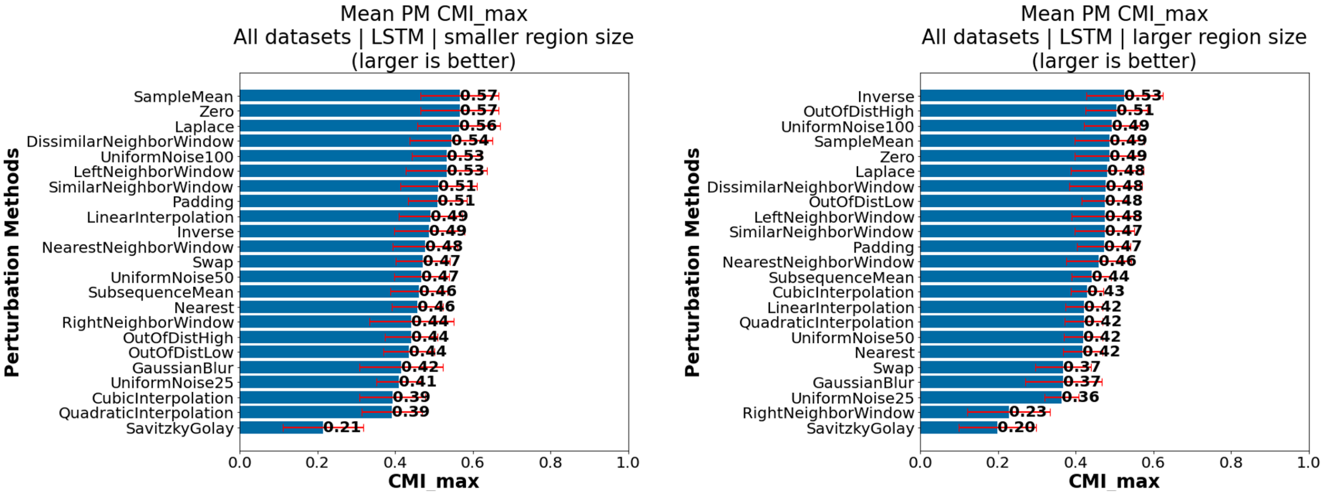

Supplementary Figure 3. Mean CMI_{max} per PM for LSTM model, across all datasets - smaller region size (left), larger region size (right).

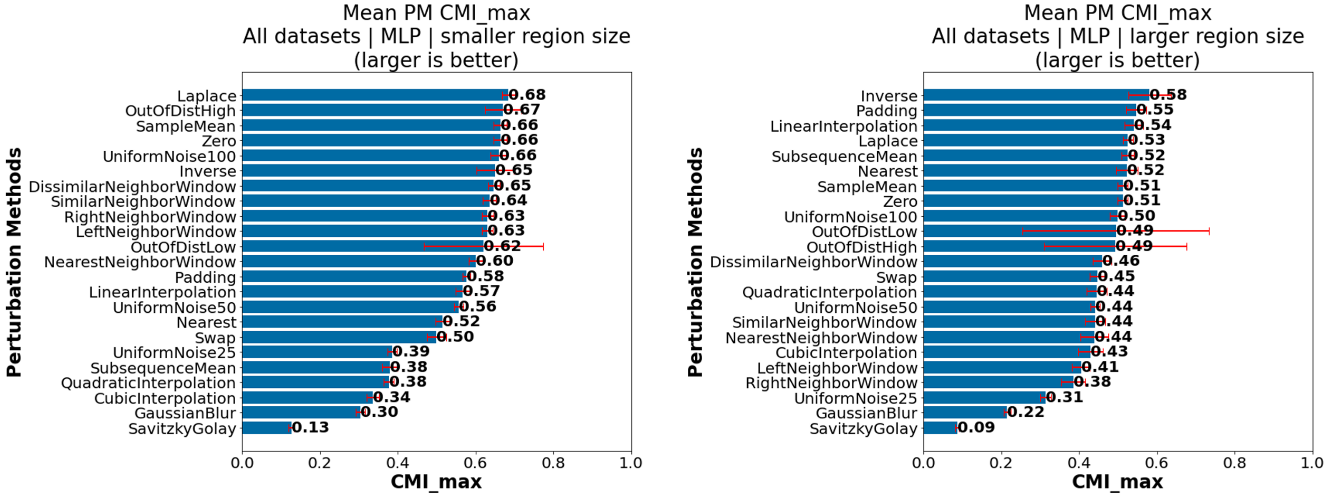

Supplementary Figure 4. Mean CMI_{max} per PM for MLP model, across all datasets - smaller region size (left), larger region size (right).

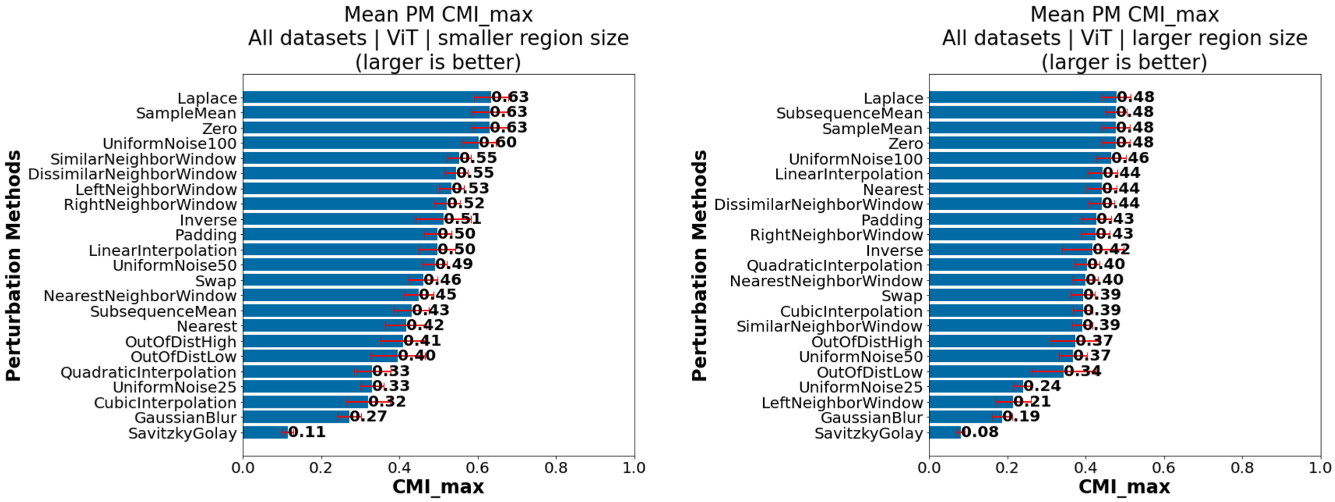

Supplementary Figure 5. Mean CMI_{max} per PM for ViT model, across all datasets - smaller region size (left), larger region size (right).

Impact of Region Size on Dataset PM Suitability

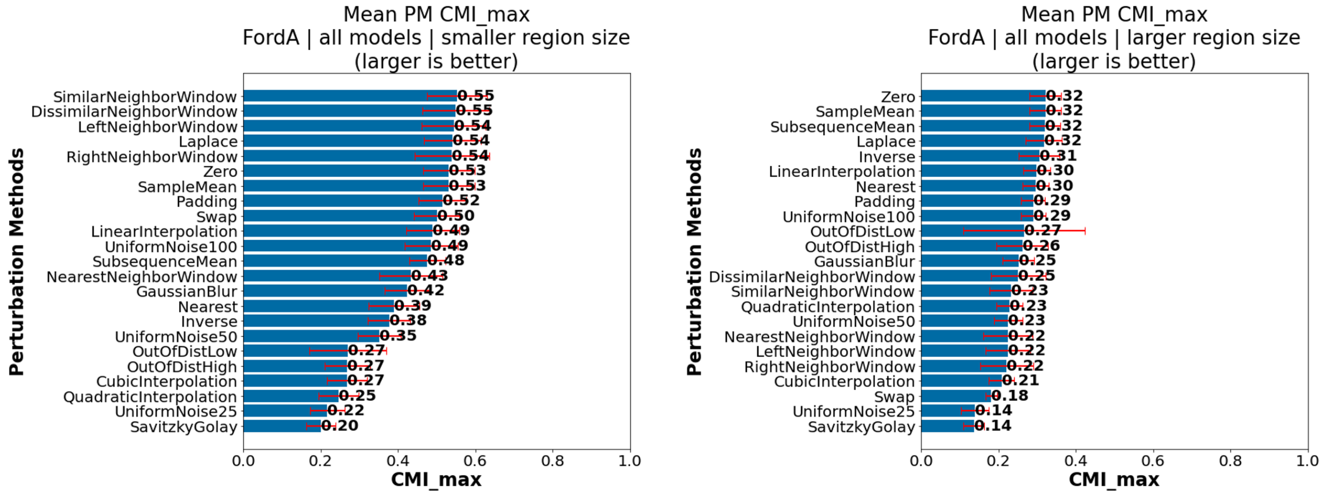

Supplementary Figure 6. Mean CMI_{max} per PM for FordA dataset, across all model architectures - smaller region size (left), larger region size (right).

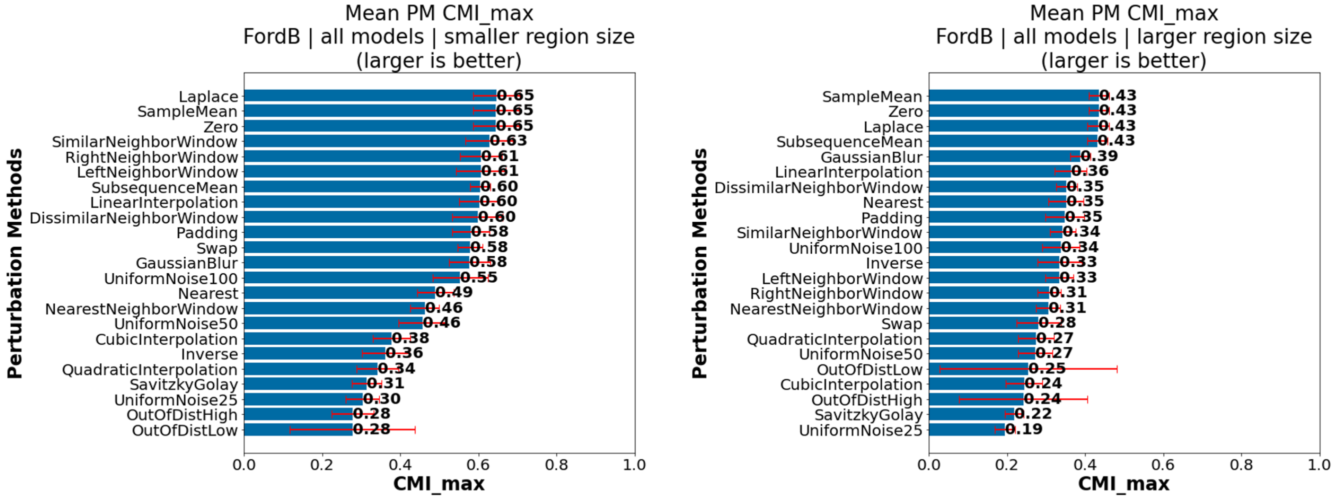

Supplementary Figure 7. Mean CMI_{max} per PM for FordB dataset, across all model architectures - smaller region size (left), larger region size (right).

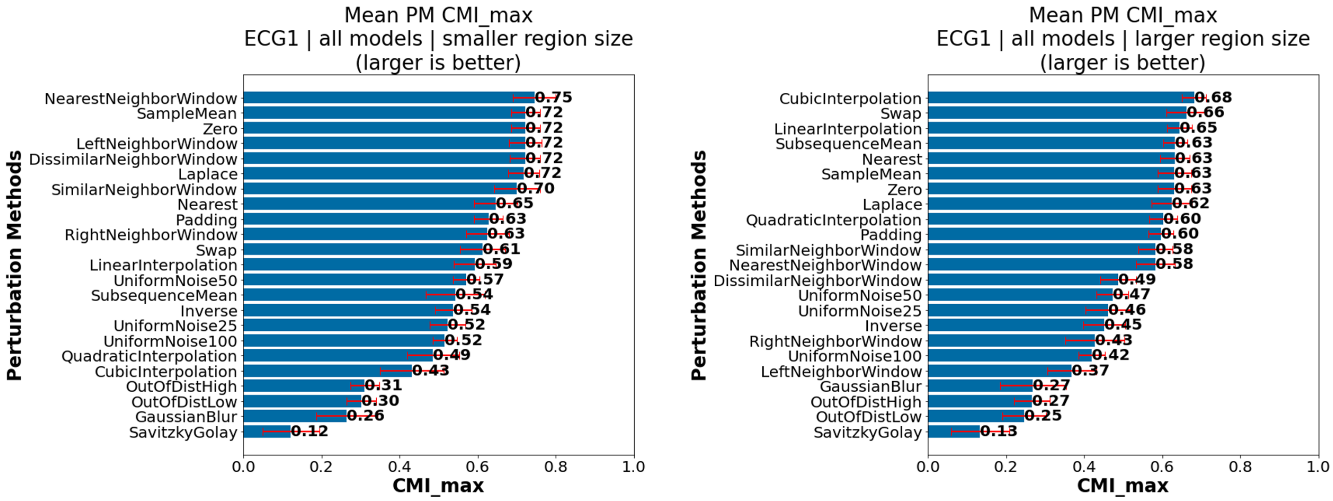

Supplementary Figure 8. Mean CMI_{max} per PM for ECG1 dataset, across all model architectures - smaller region size (left), larger region size (right).

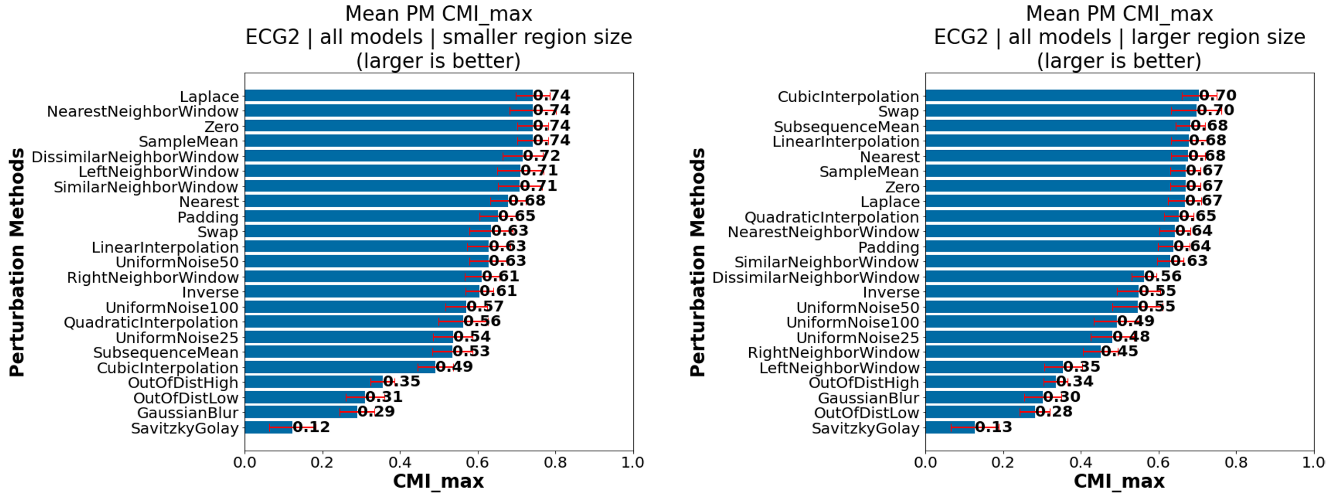

Supplementary Figure 9. Mean CMI_{max} per PM for ECG2 dataset, across all model architectures - smaller region size (left), larger region size (right).

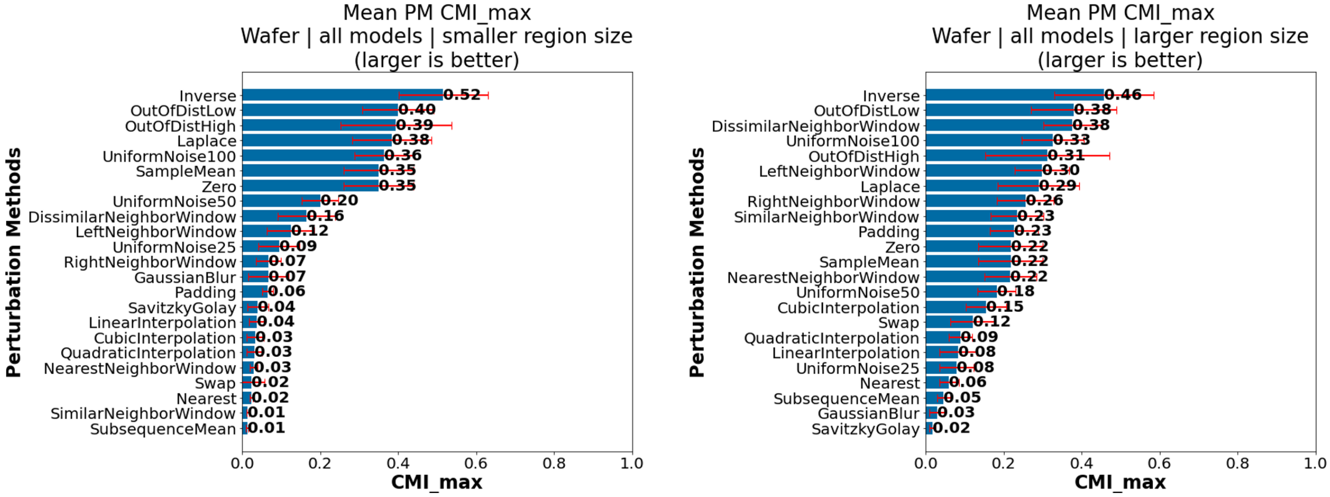

Supplementary Figure 10. Mean CMI_{max} per PM for Wafer dataset, across all model architectures - smaller region size (left), larger region size (right).
